# Multiple HA substitutions in highly pathogenic avian influenza H5Nx viruses contributed to the change in the NA subtype preference

**DOI:** 10.1080/21505594.2022.2082672

**Published:** 2022-06-05

**Authors:** Khristine Joy C. Antigua, Yun Hee Baek, Won-Suk Choi, Ju Hwan Jeong, Eun-Ha Kim, Sol Oh, Sun-Woo Yoon, Changil Kim, Eung-Gook Kim, So-Young Choi, Seung Kon Hong, Young Ki Choi, Min Suk Song

**Affiliations:** a Department of Microbiology, Chungbuk National University College of Medicine and Medical Research Institute, Cheongju, Republic of Korea; b Viral Infectious Disease Research Center, Korea Research Institute of Bioscience and Biotechnology, Daejeon, Republic of Korea; c Department of Biochemistry, Chungbuk National University College of Medicine and Medical Research Institute, Cheongju, Republic of Korea; d New Drug Development Center, Osong Medical Innovation Foundation, Cheongju, Republic of Korea; e Viruses, Korea Virus Research Institute, Institute for Basic Science (IBS)Center for Study of Emerging and Re-Emerging, Daejeon, Republic of Korea

**Keywords:** Avian influenza, clade 2.3.4, H5Nx, antigenic drift, antigenic shift, functional balance

## Abstract

Novel highly pathogenic avian influenza (HPAI) H5Nx viruses are predominantly circulating worldwide, with an increasing potential threat of an outbreak in humans. It remains largely unknown how the stably maintained HPAI H5N1 suddenly altered its neuraminidase (NA) to other NA subtypes, which resulted in the emergence and evolution of H5Nx viruses. Here, we found that a combination of four specific amino acid (AA) substitutions (S123P-T156A-D183N- S223 R) in the hemagglutinin (HA) protein consistently observed in the H5Nx markedly altered the NA preference of H5N1 viruses. These molecular changes in H5N1 impaired its fitness, particularly viral growth and the functional activities of the HA and NA proteins. Among the AA substitutions identified, the T156A substitution, which contributed to the NA shift, also dramatically altered the antigenicity of H5N1 viruses, suggesting an occurrence of antigenic drift triggered by selective pressure. Our study shows the importance of how HA and NA complement each other and that antigenic drift in HA can potentially cause a shift in the NA protein in influenza A virus evolution.

## Introduction

The highly pathogenic avian influenza (HPAI) H5N1 virus is a type of influenza virus with high zoonotic potential that causes severe disease with a high mortality rate in humans [1]. HPAI H5N1 emerged from the low-pathogenicity avian influenza (LPAI) virus in 1996 [2–5]. Since its emergence, multiple antigenically distinct sublineages have developed from this virus [2,6,7]. Although HPAI H5N1 exhibited divergence, for 12 years after it was first reported, the genes encoding the glycoprotein hemagglutinin (HA) and neuraminidase (NA) remained stable with no evidence of a major antigenic shift [8].

By 2008, H5N1 viruses belonging to clade 2 had further expanded to a third-order clade (2.3.4) while spreading geographically [9–11]. Moreover, clade 2.3.4 HPAI H5N1 suddenly began switching its long-stable N1-subtype NA to several new NA subtypes. Novel reassortant viruses, such as H5N5, H5N2, and H5N8, have been isolated and identified in poultry and migratory birds in China [1,9,11–14]. These expanded influenza viruses are collectively called “H5Nx” viruses, and they bear the same H5 Gs/Gd lineage backbone but unique NA proteins [2,15]. The resulting diversity led to clade expansion, reaching fourth-order clades (2.3.4.4) [11,15–17]. As clade 2.3.4.4 H5Nx continuously arises in the circulation, the detection of clade 2.3.4 HPAI H5N1 has gradually declined [18].

Generally, HA binds to the sialic acid receptor of the host cell for entry, while NA cleaves HA from the sialic acid receptor to release progeny virions from the host cell; thus, the interplay between HA and NA plays a vital role in functional balance [19,20]. However, sequential amino acid (AA) changes in the antigenic and/or receptor-binding regions of these glycoproteins may compromise viral fitness and to some extent facilitate host immune response escape, causing antigenic drift and/or altered receptor-binding specificity, resulting in the emergence of novel variants in the population [19–21]. Interestingly, sequential AA changes in the antigenic region, which includes T156A and D183N [1,22–25], and within (S123P, K218Q, S223 R) or distant (K82 R, N240 H) from the receptor-binding region [8,22,24–27] (H5 numbering), were observed in the HA gene between clade 2.3.4.4 H5Nx and clade 2.3.4 H5N1 viruses. While these previous findings have related mutation accumulation to antigenicity and receptor-binding activity, to date, it is poorly understood how 2.3.4 H5N1, which had remained stable for a decade, suddenly changed its NA subtype preference, which was one of the most critical evolutionary events of HPAI H5 viruses [15,24].

In this study, we aimed to determine the factors that promote the preferential substitution of the NA gene of clade 2.3.4 H5 viruses for another NA gene and demonstrate the selection that may have occurred in the natural environment. First, we evaluated the effect of substitutions in the H5 HA protein on the NA selection preference of 2.3.4 H5N1 through our developed NA selection assay, an in vitro selection assay with the application of reverse genetics. Furthermore, we demonstrated the importance of these multiple mutations in terms of viral fitness and show how their combination can contribute to the preferential change of NA protein in influenza A virus evolution.

## Methodology

### Cells and viral genes

The cell lines utilized in this study included human embryonic kidney (HEK) 293T (Korea Cell Line Bank, Seoul, Republic of Korea) and MDCK cells (ATCC, Virginia, USA). HEK 293T cells were maintained in Dulbecco’s modified Eagle’s medium (DMEM; Gibco-Invitrogen, Carlsbad, California, USA) and MDCK cells were maintained in minimum essential medium Eagle (MEM; Corning, New York, USA) supplemented with 10% (vol/vol) heat-inactivated FBS and 1% antibiotics (Gibco-Invitrogen, Carlsbad, California, USA) at 37°C under 5% CO_2_. RNA was extracted from viruses, including clade 2.2 A/environment/Korea/W149/2006 (H5N1) and clade 2.3.4.4 A/mallard/Korea/W452/2014 (H5N8) viruses. Varying internal genes (PB2, PB1, PA, NP, M, and NS) were obtained from hH1N1 (A/PuertoRico/8/1934), clade 2.2 A/environment/Korea/W149/2006 (H5N1) and clade 2.3.4.4 A/environment/Korea/W468/2014 (H5N8) viruses. The HA and NA genes of the A/duck/Anhui/1/06 (H5N1) virus and the varying NA subtypes N2 (A/duck/Jiangsu/234/2012 (H5N2)), N5 (A/duck/EasternChina/031/2009 (H5N5)), and N6 (A/duck/Guangdong/GD01/2014 (H5N6)) were artificially synthesized (Bionics, Daejeon, Republic of Korea). The directly amplified and artificially synthesized viral genes were cloned into the pHW2000 vector for reverse genetics [28,29]. The multi-basic cleavage site in all HPAI H5 HA genes was removed during PCR amplification and artificial synthesis of the genes to mitigate the safety issues.

### Reverse genetics of viruses

Sequences of 2.3.4 H5N1 and 2.3.4.4 H5Nx observed from 2005 to 2013, which were downloaded from the Influenza Research Database (www.fludb.com), were aligned using CLC Genomics Workbench 10.0.1 (Qiagen, Maryland, USA) for further analysis. Based on the molecular differences between clades 2.3.4 and 2.3.4.4, AA substitutions (K82 R, S123P, T156A, D183N, S223 R, K218Q, and N240 H into 2.3.4 H5, and the reverse substitutions into the 2.3.4.4 H5 genes) were introduced via site-directed mutagenesis using the Phusion™ Site-Directed Mutagenesis Kit (Thermo Fisher Scientific, Vilnius, Lithuania) as directed by the manufacturers. All plasmids used for viral rescue were fully sequenced to confirm the absence of unwanted mutations.

For safety, all the viruses generated possess no multibasic HA cleavage sequence becoming a non-virulent type. Viruses were rescued via reverse genetics [28,29] and propagated in 10-day-old embryonated chicken eggs (Choong Ang Vaccine Laboratories Co., Ltd., Daejeon, Republic of Korea). The HA titer was checked at 48 hr post inoculation to confirm successful rescue. All samples were subjected to RNA extraction for RT-PCR to verify correct generation of the virus as well as the lack of additional mutations. The sequences of the rescued viral genome were also analyzed to check for errors and any unwanted additional residues. All these experiments using HPAI viruses were performed in a biosafety level 3 (BSL3) laboratory at Chungbuk National University and approved by the Korea Disease Control and Prevention Agency.

### In vitro replication kinetics

Ten-day-old chicken embryonated eggs were infected with 10^2^ 50% egg infectious dose (EID_50_) and kept in a controlled incubator at 37°C. The allantoic fluid of virus-infected eggs was harvested at 12, 24, 36 and 48 hpi. The viral growth titers at each time point were determined in chicken embryonated eggs with three replicates per time point. The viral titer (log10 EID_50_/ml) was calculated and presented in a graph.

### NA selection assay

To demonstrate selection preference, 1 µg of the HA (clade 2.3.4 A/duck/Anhui/1/2006 or 2.3.4.4 A/environment/Korea/W452/2014) plasmid together with an equal amount (1 µg) of two plasmids encoding NA for selection (the N1 subtype (clade 2.3.4 A/duck/Anhui/1/06 (H5N1)) and one of the Nx subtypes (N2, N5, N6, and N8)) and an IGC of choice were transfected into a HEK293T-MDCK cell mixture for rescue [28,29]. During the NA selection assay, all the NAs used for the Nx subtype were representative of clade 2.3.4.4 novel reassortants. After careful checking for correct sequences of rescue supernantants, a 100 ul volume of rescue supernatants (R0) were harvested 5 days after transfection and infected to 10-day-old embryonated chicken eggs and/or MDCK cells for the first passage (P1). 10^2^TCID_50_ of P1 viruses were subsequently passaged to the eggs and/or MDCK cells (P2). Forty-eight hours post-inoculation, the allantoic fluid and/or MDCK supernatant were harvested and treated with DNase (Qiagen, Maryland, USA) during RNA extraction with the RNeasy Mini RNA Extraction Kit (Qiagen, Maryland, USA). After amplification, cDNA samples from the rescue supernatant (R0), passage one (E1/C1), and passage two (E2/C2) analyzed by real-time (RT) quantitative (q)PCR (Biorad CFX96 Touch Real-Time PCR 3.0, California USA). To lessen amplification bias and increase the accuracy of relative detection, four sets of primers for each subtype were specifically designed and used for the NA selection assay. Real-time PCR was performed using 5 pmol of specifically designed primer sets (Table S5). The cycling conditions included initial denaturation at 95°C 3 min; 40 cycles of denaturation at 95°C 30 s, annealing at 58°C 15 s, and elongation at 72°C 15 s; and a final elongation step at 72°C 15 min. Additionally, following the last amplification cycle, the reaction temperature was rapidly increased to 95°C 10 s, then decreased to 65°C for 5 s, and finally slowly increased to 95°C at a rate of 0.5°C/sec, with continuous fluorescence monitoring. In this assay, three IGCs (selected from human H1N1, clade 2.2, and 2.3.4.4 IGCs) were utilized in the rescue platform to confirm any possible unknown contribution from internal genes other than the HA gene. All the primers used for real-time PCR are listed in Table S5.

### Hemagglutinin receptor binding assay

The receptor-binding specificity was determined following a modified HA receptor-binding assay previously described [30]. Briefly, 0.2 ml of a working solution of 10 mg/ml fetuin in PBS was used to block a 96-well microtiter plate. After incubation overnight at 4°C the plates were washed with distilled water and allowed to air dry before the addition of 0.01 ml of a solution containing 32 hemagglutination units (HAU) of virus in Tris-buffered saline (TBS). In addition, 0.01 ml of TBS was utilized as a negative control. The plate was then incubated overnight and washed three times with cold PBS. The plate was then blocked with a 1% BSA solution and incubated again overnight. After washing the plate with ice-cold PBS-Tween (PBST), the plates were incubated at 4°C for 4 hr with serially diluted 0.01 ml volumes of 3'-sialyllactose-polyacrylamide (PAA)-biotin 6'-sialyllactose-PAA-biotin (Glycotech Co., Maryland, USA) glycan diluted in 1% BSA in PBS with 0.001% Tween 80 and 1 µM zanamivir at 4°C for 4 hr. After washing with ice-cold PBST, 0.01 ml of a horseradish peroxidase solution was added to each well for detection. After 1 hr of incubation in the dark at 37°C and six washes with PBST, peroxidase activity was detected using 33’,55’-tetramethylbenzidine (TMB), and the reaction was stopped by adding 2 M H_2_SO_4_. The absorbance (optical density) was measured at a wavelength of 450 nm using a Synergy™ HTX Multi-Mode Microplate Reader (Biotek, Vermont, USA). To reflect the HA binding constants representing the binding avidities between HA and the glycans, the Kd values were determined using nonlinear regression analysis in GraphPad Prism 9.1 (La Jolla, California, USA). The Kd values were then calculated using the formula of Y=Bmax*X/(Kd+X) + NS*X + Background, wherein Bmax represents the maximum specific binding; NS value as the slope of nonspecific binding in Y units divided by X units; while the amount of nonspecific binding is measured without the added glycans were presented as background. Appropriate negative controls (without the addition of glycans) were included, and all assays were performed in triplicate.

### NA activity and enzyme kinetics assay

The NA activity of each virus was determined using an NA-Star Influenza Neuraminidase Inhibitor Resistance Detection Kit (Applied Biosystem, CA, USA) following the manufacturer’s protocol. The signal was measured in duplicate using a Synergy HTX multimode microplate reader at 492 nm using a Synergy™ HTX Multi-Mode Microplate Reader (Biotek, Vermont USA). All results are presented as the means from two or three independent experiments. The NA enzymatic properties of the recombinant viruses were determined following the protocol of Marathe et al [31]. Briefly, the recombinant viruses were serially diluted twofold up to 1:1,024 to identify the viral dilutions that generated a proportional increase in the concentration of 4-methylumbelliferone after incubation with the 2'-(4-methylumbelliferyl)-α-D-*N*-acetylneuraminic acid (MUNANA) substrate [31]. The enzymatic reactions were performed in 96-well flat-bottom black plates incubated at 37°C in a total volume of 100 μl (25 μl of appropriately diluted virus, 25 μl of enzyme buffer (32.5 mM 2-(*N*-morpholino) ethane sulfonic acid (MES) in pH 6.5 and 4 mM CaCl2), 50 μl of MUNANA solution per well) with different final concentrations of MUNANA, ranging from 0 to 1,000 μM. The reaction plates were shaken for 30 s and transferred to a prewarmed Synergy™ HTX Multi-Mode Microplate Reader (Biotek, Vermont, USA). The fluorescence was monitored every 60 s for 60 min at 37°C using excitation and emission wavelengths of 360 nm and 460 nm, respectively. To determine the enzyme kinetic parameters, such as the maximum velocity (Vmax), Michaelis constant (Km), and catalytic efficiency (Kcat), enzyme kinetic data readings were analyzed by fitting the data to the Michaelis–Menten equation using nonlinear regression in GraphPad Prism 9.1 (La Jolla, California, USA).

### Hemagglutination inhibition assay

Antibody titers were determined by an HI assay as described elsewhere [32,33]. The antibodies utilized in this study were specific antibodies for influenza antigen A/Anhui/1/05 (H5N1) IBCDC-RG6 (National Institute for Biological Standards and Control, England, United Kingdom) and laboratory-sourced serum elicited from ferret immunized with influenza HA antigen of A/gyrfalcon/Washington/41,088-6/2014 (H5N8) IDCDC-RG43A, previously described and tested by Jeong et al., (2019) [34]. As previously described [33], to remove nonspecific HA activity, the anti-sera were treated with receptor-destroying enzyme (RDE, Denka Seiken, Japan) for 18 hr. The serum was diluted 1:10 PBS or allowed to have hemadsorption against a 10% RBC solution for 30 min at room temperature. To perform the HI assay, a serial twofold dilution of 25 µl of RDE-treated sera in PBS was performed in a V-bottom 96-well microtiter plate. Then, the plate was incubated with equal volumes of 4–8 HAU of the virus/50 µl at room temperature for 30 min to allow the serum and virus to interact. The HI titers were then observed after 30 min of incubation with a 0.5% chicken RBC solution and recorded as the reciprocal of the highest dilution of the serum that did not result in agglutination of the erythrocytes.

### Generation of an antigenic map

An antigenic cartography map was constructed as previously described [35]. Each entry in the HI table was normalized and analyzed by low-rank matrix completion. Each unit of the antigenic distance corresponded to a twofold change in HI titer. Multidimensional scaling was used to project viruses onto a two-dimensional map by minimizing the sum of the squared error between the map distance and the antigenic distance. Using R version 3.5.3 [36], we calculated the antigenic differences by calculating the mean Euclidean distance ($d{\left(p,q\right)}^{2}={\left(q_{1}-p_{1}\right)}^{2}+{\left(q_{2}-p_{2}\right)}^{2})$, where we defined *p* and *q* as the numerical difference of the mean coordinates of the *Rg* virus group identified in the Euclidean plane.

### Homology modeling

The crystal structures of the HA protein from the H5N1 virus (A/turkey/Turkey/1/2005) in complex with 6' sialyl (*N*-acetyllactosamine) (SLN) (Protein Data Bank (PDB) accession no. 4BH0) [37] and NA of A/tern/Australia/G70C/1975 (H11N9) (PDB accession no. 6CRD) [38] were used as templates for simulation of the HA and NA interactions. To simulate the protein–protein complex, we utilized the ZDOCK server [39] to select an appropriate model for the docking complex of HA with NA as a receptor. For homology modeling, the WT and mutant HA were three-dimensionally aligned with the crystal structures via the SWISS-MODEL server [40]. Images were generated using PyMOL (http://www.pymol.org/).

### Statistical analysis

The Mean ∆Ct values and glycoprotein-binding activities were analyzed using Two-way ANOVA with Tukey’s honest test for multiple comparison posttest. Multiple *T*-test comparison was used to analyzed the log_10_-transformed viral titers with *P-*value of ≤0.05 considered significant. The mean NA enzymatic activity values utilized one-way ANOVA and Tukey’s honest test for multiple comparison posttest with significant *P*-value of ≤0.05.

## Results

### The H5 gene is critical for preferential selection of the NA gene

**Figure 1.**
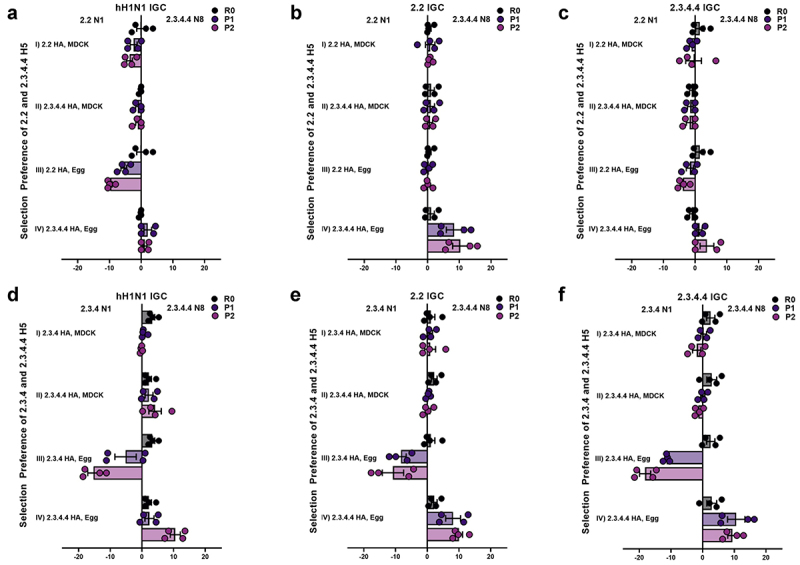
**Neuraminidase (NA) selection preference of H5 viruses of clades 2.2, 2.3.4 and 2.3.4.4**. an in vitro NA selection assay was utilized by applying reverse genetics to the wild-type (WT) 2.2, 2.3.4, and 2.3.4.4 H5 viruses, combined with varying internal gene cassettes (IGCs) of different viral origins and a selection of two NA genes, namely, N1 and N8. The NA selection was compared between 2.2 H5 and 2.3.4.4 H5 (a, b, and c) and between 2.3.4 H5 and 2.3.4.4 H5 (d, e, and f) in Mardin-Darby canine kidney (MDCK, i-ii) cells and chicken embryonated eggs (iii-iv). The mean δct values represent the preference for the representative NA subtype, namely, N1 or N8. The mean δct values of a minimum four determinations are presented in each graph. The error bars represent standard errors. R0: rescue supernatant; P1: cell or egg passage 1; P2: cell or egg passage 2; IGC: internal gene cassette; Ct: cycle threshold.

To determine the factors (HA gene, internal gene cassettes (IGCs), and host) that promote the preferential substitution of the NA gene of clade 2.3.4 H5 viruses for another NA gene and demonstrate the selection that may have occurred in the natural environment, a novel in vitro NA selection assay was utilized by applying reverse genetics to the wild-type (WT) H5 viruses, combined with varying the IGCs of different viral origins and selection of two NA genes, namely, N1 and N8 (Figure S1). To mimic hosts and the possible reassortment occurring in the natural environment, the rescued re-assortant (R0) in the assay was passaged twice in chicken embryonated eggs or Madin-Darby canine kidney (MDCK) cells (P1, P2). Primarily, the NA gene selection assay was performed to investigate whether the shift of the H5N1 NA gene to other NA subtypes, including N8, was affected by the HA gene (clades 2.2, 2.3.4, and 2.3.4.4) and/or IGCs (human H1N1, clades 2.2 and 2.3.4.4 H5) in MDCK and chicken embryonated eggs. We determine host impact on NA selection when performed with passaging in MDCK cells. However, preferential selection of the clade 2.2, 2.3.4 N1, or 2.3.4.4 N8 gene by the 2.2, 2.3.4, and 2.3.4.4 H5 genes was not observed in MDCK cells (Figure 1(a–f, i and ii)). On the other hand, the determination in eggs showed that although the presence of the clade 2.2 H5 gene led to preferential selection of the 2.2 N1 gene over the 2.3.4.4 N8 gene (Figure 1(a–c, iii and iv)), the presence of the clade 2.3.4 H5 gene led to the clear selection of the 2.3.4 N1 gene over the 2.3.4.4 N8 gene in all IGCs (Figure 1(d–f, iii and iv). The presence of the clade 2.3.4.4 H5 gene led to preferential selection of the 2.3.4.4 N8 gene over both the 2.2 (Figure 1(a–c, iii and iv)) and 2.3.4 (Figure 1(d–f, iii and iv)) N1 genes regardless of the IGCs during egg passage. These results show how the H5 gene plays a central role, among the tested factors (NA gene component, IGC, and host factors), in the alteration of the NA gene in the recent circulation of the clade 2.3.4.4 HPAI H5N8 specifically among the avian host.

### Prototype of 2.3.4.4 H5Nx was reported after accumulation of the specific substitutions

**Figure 2.**
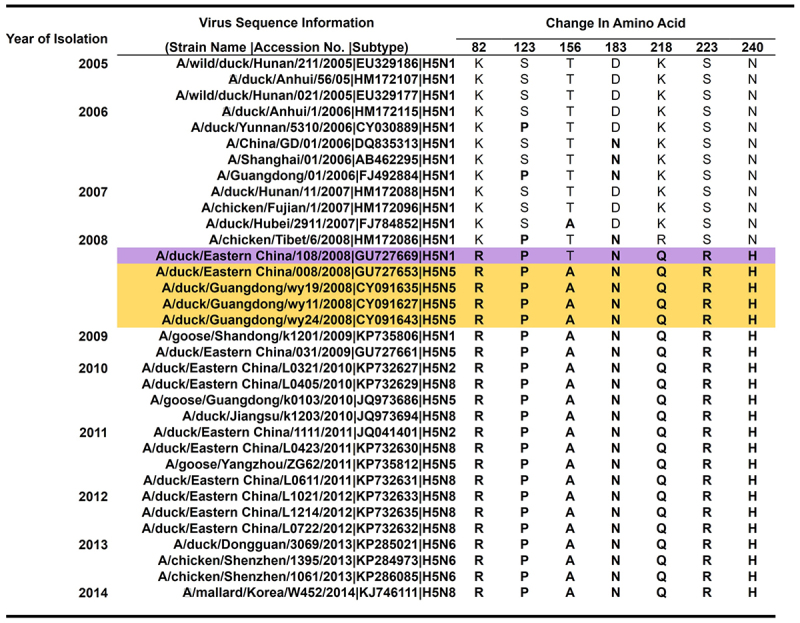
**Sequence alignment of sequentially accrued substitutions in the H5 gene from 2005 to 2014**. Haemagglutinin (HA) sequences of clade 2.3.4 and 2.3.4.4 viruses from 2005–2014 were downloaded from the influenza research database (IRD) and aligned chronologically using CLC genomics workbench 10.0.1. The seven identified conserved residues (82, 123, 156, 183, 218, 223, and 240) between 2.3.4 and 2.3.4.4 are highlighted in pink. The first protypes of 2.3.4.4 viruses isolated with the seven amino acid (AA) substitutions in their H5 genes are highlighted in yellow. Dots: unchanged residues. The reference WT viruses utilized in the study for 2.3.4 H5 (*) (A/duck/anhui/1/2006) and 2.3.4.4 H5 (**) (A/mallard/korea/w452/2014) are marked with asterisks.

After determining the role of the H5 gene in the preferential selection of the NA gene, we further investigated the genetic difference between clade 2.3.4 and 2.3.4.4 H5 genes. First, we compared the AA sequences of clade 2.3.4 and 2.3.4.4 H5 by sequence alignment, revealing seven highly conserved AA substitutions in the H5 globular head (K82 R, S123P, T156A, D183N, S223 R, K218Q, and N240 H in H5 numbering), consistent with previous studies [24,25]. By chronological alignment, we revealed the molecular events that may have occurred before the NA alteration of clade 2.3.4 and the emergence of 2.3.4.4 H5Nx viruses (Figure 2). In 2008, six of the seven conserved mutations, K82 R-S123P-D183N-Q218K-S223 R-N240 H in the H5 protein, were observed in the sequence of the 2.3.4 H5N1 virus (A/Duck/Eastern China/108/2008) (Figure 2, highlighted in purple). Within the same year, the AA substitution T156A in combination with the six mutations was first observed among the H5N5 isolates identified during live poultry surveillance in China [9,12,41]. Interestingly, the first recorded prototypes of clade 2.3.4.4 viruses were A/Duck/Eastern China/008/2008 (H5N5) [9], A/Duck/Guangdong/wy11/2008 (H5N5) [12], A/Duck/Guangdong/wy19/2008 (H5N5) [12], and A/Duck/Guangdong/wy24/2008 (H5N5) [12] (highlighted in yellow). Moreover, the genetic variations analyzed also revealed the sequential single-nucleotide polymorphism (SNP) frequency between clade 2.3.4 (n = 189) and 2.3.4.4 (n = 69) sequences from 2005 to 2013 with 100% alteration in the 7 AA positions in sequences collated after 2008 (Table S1). The result of genetic comparison between the 2.3.4 and 2.3.4.4 H5 HA genes suggests that these observed mutations introduced into 2.3.4 H5 may have contributed to the change in the preferential NA selection of 2.3.4 H5 viruses and the divergence of clade 2.3.4.4 H5.

### Specific substitutions in the H5 gene are the key determinants of preferential NA gene selection in clade 2.3.4 and 2.3.4.4 H5 viruses

**Figure 3.**
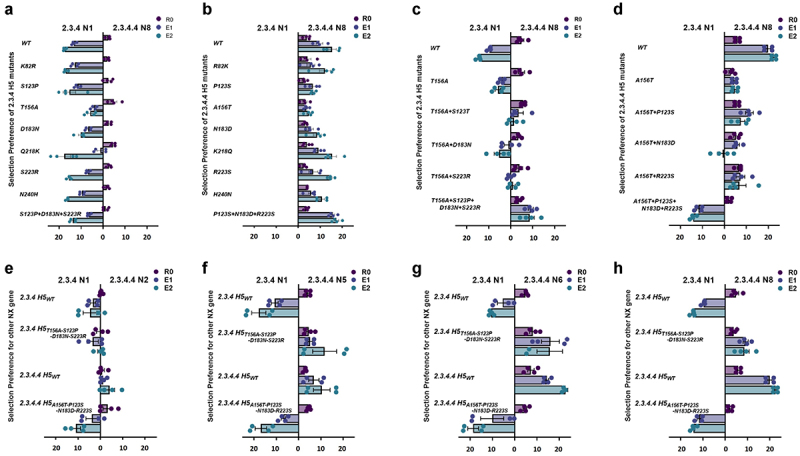
**Assessment of single or combined AA substitutions in the 2.3.4 H5 gene and its reverse substitutions in 2.3.4.4 H5 for preferential NA gene selection**. Based on the sequence alignment, NA selection was carried out via single AA substitution (AAS) of the conserved mutations between 2.3.4 (a) and 2.3.4.4 (b) H5 viruses. With the observed significant reduction in fold change preference and as the last mutation introduced to 2.3.4 H5 before 2.3.4.4 emergence, T156A in combination with mutation/s, S123P/D183N/S223 R in 2.3.4 (c) and its reverse substitutions in 2.3.4.4 (d), H5 viruses were determined to show preferential NA selection between 2.3.4 N1 and 2.3.4.4 N8. With combined substitutions in the 2.3.4 and 2.3.4.4 H5 genes, NA selection was performed for the 2.3.4 and 2.3.4.4 WT versions and the 123-156-183-223 residue mutation combination against various 2.3.4.4 NA subtypes of N2 (e), N5 (f), N6 (g), and N8 (h). The mean δct values represent the preference for the representative NA subtype, N1 or N2, N5, N6, and N8. The mean δct values of three determinations are presented in each graph with error bars as standard errors. R0: rescue supernatant; E1: egg passage 1; E2: egg passage 2; Ct: cycle threshold.

To determine the impact of the conserved residues identified in 2.3.4 and 2.3.4.4 H5 on the change in preferential NA selection, we further investigated this phenomenon by introducing substitutions (K82 R, S123P, T156A, D183N, S223 R, K218Q, and N240 H) individually or in combination into the clade 2.3.4 H5 gene and vice versa into the 2.3.4.4 H5 gene in viruses containing the 2.3.4.4 IGC in eggs via the NA selection assay (Figure 3(a,b)). Although none of the individual or combined substitutions altered the NA selection preference, T156A, which causes loss of *N*-glycosylation [42] in the 2.3.4 H5 gene and vice versa in the 2.3.4.4 H5 gene, dramatically reduced the fold change preference of the mutant viruses after 2 passages (E2) compared to their respective WT H5 gene (Figure 3(a,b)). Subsequently, the AA substitutions S123P, D183N, and S223 R, which are known to be associated with HA receptor-binding activity and antigenicity [8,22,27,43], were introduced together with the last introduced mutation, T156A, in the field [41] (Figure 3(c,d)) into the 2.3.4 H5 gene and vice versa into the 2.3.4.4 H5 gene. Although a dramatic reduction in the 2.3.4 N1 gene preference by the 2.3.4 H5 gene was observed when any two of these mutations were combined, complete alteration was not observed (Figure 3(c)). Notably, when the T156A substitution was combined with the three other AA substitutions (T156A-S123P-D183N-S223 R) in the 2.3.4 H5 gene, the preferential selection of the N1 gene was completely altered to the N8 gene (Figure 3(c)). NA preference alteration was also observed when the reverse substitutions (A156T-P123S-N183D-R223S) were introduced into the 2.3.4.4 H5 gene (Figure 3(d)). Collectively, the T156A substitution in the H5 gene of the clade 2.3.4 virus combined with three additional substitutions played a key role in the altered preference for N1 versus N8.

### The four identified mutations also showed preferential selection for the N5 and N6 subtypes

There have been various 2.3.4.4 H5Nx viruses in circulation, including H5N2, H5N5, H5N6, and H5N8 (Figure 2). To verify whether the combination of the four substitutions found (T156A-S123P-D183N-S223 R) in the 2.3.4 H5 gene could also lead to preferential selection of the other 2.3.4.4 NA genes, including N2, N5, and N6, than N1, an NA selection assay was performed in embryonic chicken eggs. The WT 2.3.4 H5 gene led to the selection of the N1 gene over the other 2.3.4.4 NA genes, while the WT 2.3.4.4 H5 gene led to preferential selection of N2, N5, N6, or N8 over N1 during 2 passages (Figure 3(e–h)). Notably, the T156A-S123P-D183N-S223 R substitutions in the 2.3.4 H5 gene completely altered the selection of N1 in favor of the other NA genes (Figure 3(f–h)), except for the N2 gene, for which a change was not clearly observed (Figure 3(e)). Moreover, the reverse substitutions in the four residues in the 2.3.4.4 H5 gene clearly altered the preferential selection of the N2, N5, N6, and N8 genes in favor of the N1 gene (Figure 3(e–h)). This result demonstrated that the combined T156A-S123P-D183N-S223 R substitutions in the 2.3.4 H5 gene enhanced its compatibility with other NA genes, such as those of the recently circulating 2.3.4.4 H5 viruses.

### Introduction of residue combinations at positions 156-123-183-223 in the H5 gene alters viral growth and fitness

**Figure 4.**
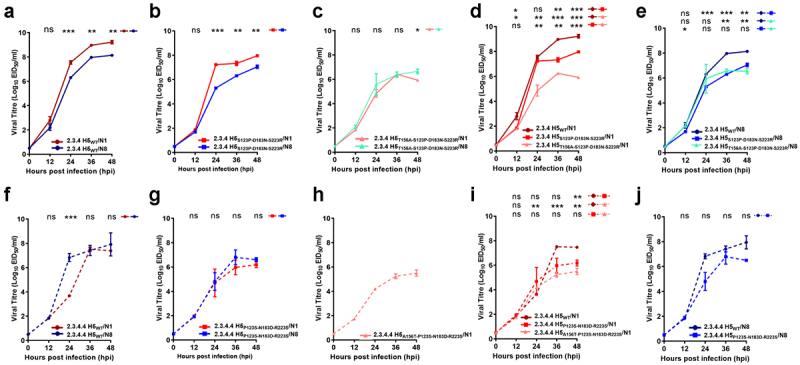
**Invitro replication kinetics of 2.3.4 and 2.3.4.4 H5 WT viruses and viruses withHA gene mutation combinations**. Based on the natural sequence of occurrence, reverse genetics (*Rg*) viruses, including 2.3.4/2.3.4.4 H5_WT_, 2.3.4/2.3.4.4 H5_S123P-D183N-S223 R_, and 2.3.4/2.3.4.4 H5_T156A-S123P-D183N-S223 R_with N1 or N8, were selected and evaluated for viral growth. Comparison between 2.3.4 H5 *Rg* viruses with N1 and N8(a, b, and c) and within 2.3.4 H5 *Rg* viruses with N1 (d) and N8 (e) is shown. Additionally, comparison between 2.3.4.4 H5 *Rg* viruses with N1 and N8 (F, G, and H)and within 2.3.4.4 H5 *Rg* viruses withN1 (i) and N8 (j) is shown. Eggs were infected with 10^2^ 50% egg infectious dose (Eid_50_), the allantoic fluid was harvested at 12,24, 36 and 48 hr post infection (hpi), and the EID_50_ viral titers were determined. The results presented are the mean values of 3 repetition of performed assays. The error bars represent standard errors. Significant results according to the calculated *P*-values (*, *P≤*0.05; **, *P≤*0.005;***, *P≤*0.0005; ns, *P* >0.05) are indicated.

As the T156A residue was shown to be acquired after the S123P-D183N-S223 R substitutions in the 2.3.4 H5 protein before the emergence of the first 2.3.4.4 H5N5 virus as shown in Figure 2 and Guo et al., (2017) [41], and that it plays a key role in preferential NA selection (Figure 3). We next aimed to determine the impact of these residue combinations on viral fitness by evaluating the replicative kinetics of the 2.3.4 H5_WT_ and re-assortant viruses with historical mutations in embryonated chicken eggs. The virus with WT 2.3.4 H5 and N1 (2.3.4 H5_WT_/N1) exhibited significantly (P ≤ 0.005) better viral titers over 48 hr than the virus with WT 2.3.4 H5 and N8 (2.3.4 H5_WT_/N8) (Figure 4(a)). A virus with the mutation combination S123P-D183N-S223 R in 2.3.4 H5 in combination with N1 (2.3.4 H5_S123P-D183N-S223 R_/N1) exhibited less efficient viral growth than 2.3.4 H5_WT_/N1 within 36–48 hr post-infection (hpi) (P ≤ 0.005–0.0005) (Figure 4(d)) but still grew more efficiently than 2.3.4 H5S123P-D183N-S223 R/N8 (P < 0.005) (Figure 4(b)). The 2.3.4 H5_T156A-S123P-D183N-S223 R_ viruses, regardless of the associated NA, exhibited significantly lower viral growth kinetics than their respective 2.3.4 H5 WT after 36 hpi (P ≤ 0.05) (Figure 4(d–e)). Notably, the 2.3.4 H5_T156A-S123P-D183N-S223 R_/N8 virus exhibited viral growth similar to or better (P ≤ 0.05, 48 hpi) than that of the 2.3.4 H5_T156A-S123P-D183N-S223 R_/N1 virus (Figure 4(c)). Taken together, these data indicate that compared to the WT version, 2.3.4 H5 carrying T156A-S123P-D183N-S223 R showed impaired viral replicative fitness with N1, but the mutation combination provided the virus with better growth properties with the selection of another NA subtype over N1.

Although there is no reported evidence of 2.3.4.4 H5 viruses with the reverse mutations (P123S-N183D-R223S/A156T-P123S-N183D-R223S) in their H5 gene existing in nature, for comparative purposes, we also determined their growth properties via in vitro replication kinetics assays. The 2.3.4.4 H5_WT_/N8 virus grew better of almost 1,000-fold higher than the 2.3.4.4 H5_WT_/N1 at 24 hpi (P ≤ 0.0005; Figure 4(f)) denoting its growth advantage at early time point. However, the findings for 2.3.4.4 H5 viruses remain inconclusive because of the several failed attempts to recover and regenerate the 2.3.4.4 H5_A156T-P123S-N183D-R223S_/N8 (Figure 4(h)). These findings may suggest that the combination of these 4 reverse substitutions alone is not sufficient to result in reversion and efficient generation of the virus with the N8 gene in the recent 2.3.4.4 H5 HA gene background.

### T156A in the presence of the 3 other substitutions in 2.3.4 H5 with N8 restored the HA and NA balance to a level better than that of the 2.3.4 H5_WT_/N1 virus and comparable to that of 2.3.4.4 H5_WT_/N8

**Figure 5.**
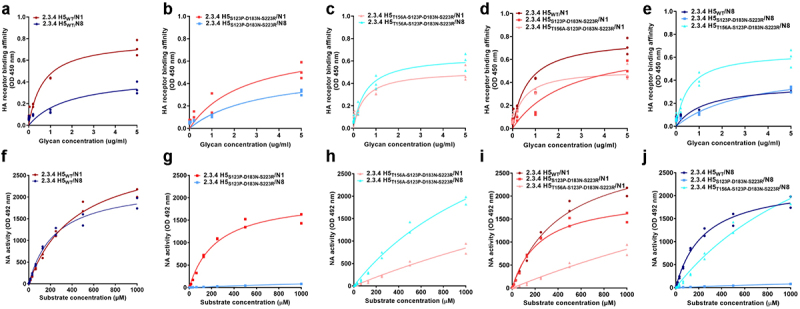
**Glycoprotein activities of the 2.3.4 H5 WT virus and viruses with H5 gene mutation combinations**. Based on the natural sequence of occurrence, reverse genetics (*Rg*) viruses, including 2.3.4/2.3.4.4 H5_WT_, and 2.3.4/2.3.4.4 H5 combination mutants with N1 or N8, were selected and evaluated for their HA and NA activities. In addition, the HA receptor binding affinity of 2.3.4 H5_WT_, 2.3.4 H5_S123P-D183N-S223 R_, and 2.3.4 H5_T156A-S123P-D183N-S223 R_ with N1 or N8 was measured using α2,3 biotinylated glycans using HA direct binding assay read at OD 450 nm. A comparison of HA receptor binding affinity between 2.3.4 H5 *Rg* viruses with N1 and N8 (a, b and c) and within 2.3.4 H5 *Rg* viruses with N1 (d) and N8 (e) is shown. NA activity of 2.3.4 H5_WT_, 2.3.4 H5_S123P-D183N-S223 R_, and 2.3.4 H5_T156A-S123P-D183N-S223 R_ with N1 or N8 was also determined through a chemiluminescence assay read at OD 492 nm. Comparison of NA activity between 2.3.4 H5 *Rg* viruses with N1 and N8 (F, G, and H) and within 2.3.4 H5 *Rg* viruses with N1 (I) and N8 (J) is shown. The results presented are the mean values of a minimum of 3 repetitions of performed assays.

To demonstrate the possible impact of the combined residues T156A-S123P-D183N-S223 R in the H5 gene on the HA and NA activity of the virus, we determine the effect of mutations introduced in the H5 gene as well as the impact of NA alteration on the binding activities of each glycoprotein, we measured the binding affinity of the WT and reverse genetics (*Rg*) viruses to the α2,3-sialic acid receptor, which is predominant in avian hosts, using a solid-phase receptor-binding assay [30]. Here, we assumed that the most balanced virus among the set tested was 2.3.4 H5_WT_/N1 or 2.3.4.4 H5_WT_/N8, which has circulated in nature. Thus, in the following tests, we compared the results between *Rg* viruses with mutations in H5 and the respective WT versions of 2.3.4 (Figure 5) or 2.3.4.4 viruses (Figure S2). Notably, 2.3.4 H5_WT_/N1 exhibited higher HA receptor binding activity of Kd = 0.486 ± 0.25 µM than 2.3.4 H5_WT_/N8 showed Kd = 1.368 ± 0.14 µM (Figure 5(a) and Table S2). The presence of three mutations (S123P-D183N-S223 R) in the 2.3.4 H5 gene had more impact on the binding properties of N1 than N8 *Rg* viruses (Figure 5(b,d,e). After the additional introduction of the T156A mutation, 2.3.4 H5_T156A-S123P-D183N-S223 R_/N1 (Kd = 0.342 ± 0.13 µM) and 2.3.4 H5_T156A-S123P-D183N-S223 R_/N8 (Kd = 0.387 ± 0.21 µM) conferred similar HA receptor binding affinities (Figure 5(c) and Table S2). Notably, 2.3.4 H5_T156A-S123P-D183N-S223 R_/N8 also showed HA affinity comparable with that of 2.3.4.4 H5_WT_/N8 (Kd = 0.387 ± 0.21 VS 0.432 ± 0.04 µM, respectively) (Table S2, Figure 5(c) and S2A). The binding affinity of all single mutants of 2.3.4 H5/N1 was higher than those of single mutants of 2.3.4 H5/N8 (Figure S3A-D). Additionally, the receptor-binding affinities toward mammalian hosts, measured using α-2,6 sialic acid receptors, were not changed in any of the viruses in this study (Figure S4). Overall, the introduction of the T156A mutation in the presence of the 3 other mutations in the 2.3.4 H5 virus with N8 significantly restored its HA activity to a level comparable activity to that of 2.3.4.4 H5_WT_/N8 when combined with N8.

Next, we characterized the effects of AA substitutions in HA on the NA genes. The 2.3.4 H5_WT_/N1 virus showed slightly better NA activity than 2.3.4 H5_WT_/N8 (Figure 5F). Although both 2.3.4 H5_S123P-D183N-S223 R_/N1 and 2.3.4 H5_S123P-D183N-S223 R_/N8 showed reduced NA activities compared to those of 2.3.4 H5_WT_/N1 and H5_WT_/N8, the 2.3.4 H5_S123P-D183N-S223 R_/N8 virus displayed almost no NA activity (Figure 5(f,g)). Notably, the introduction of T156A restored the NA activity of 2.3.4 H5 _T156A-S123P-D183N-S223 R_/N8 (Figure 5(i,j)), while the 2.3.4 H5_T156A-S123P-D183N-S223 R_/N1 virus dramatically decreased NA activity compared to that of the 2.3.4 H5_WT_/N1 virus (Figure 5(i)), which is lower than that of 2.3.4 H5 _T156A-S123P-D183N-S223 R_/N8 (Figure 5(h)). The NA enzymatic activity measured using a fluorogenic enzyme kinetic assay [31] revealed that 2.3.4 H5_WT_/N1 (Kcat/Km = 5.24 ± 0.23) was decreased in 2.3.4 H5_T156A-S123P-D183N-S223 R_/N1 (Kcat/Km = 4.95 ± 0.27), while the enzymatic activity of 2.3.4 H5_T156A-S123P-D183N-S223 R_/N8 was significantly improved (Kcat/Km = 6.55 ± 0.01) compared to that of 2.3.4 H5_T156A-S123P-D183N-S223 R_/N1 (Table S2 and Figure S5), suggesting the occurrence of improved progeny release and spread when 2.3.4 H5_T156A-S123P-D183N-S223 R_ was combined with N8. Overall, the results indicate that T156A in the presence of the 3 other substitutions in the 2.3.4 H5_T156A-S123P-D183N-S223 R_/N8 virus restores the HA-receptor binding affinity and NA activity and confers an HA and NA balance better than that of 2.3.4 H5_T156A-S123P-D183N-S223 R_/N1 and comparable to that of 2.3.4.4 H5_WT_/N8.

### T156A substitution dramatically changes the antigenic properties of the 2.3.4 H5 viruses

To explore whether the substitutions found are related to host immune escape, we then proceeded to investigate whether the single and/or combined mutations altered innate antigenic properties. We tested the reactivity pattern of the *Rg* viruses with anti-sera raised against specific 2.3.4 and 2.3.4.4 H5N8 HA proteins using a hemagglutination inhibition (HI) assay [34]. The *Rg* viruses belonging to clade 2.3.4 without the T156A substitution but with other single mutations or the S123P-D183N-S223 R combined mutation exhibited the least change in inhibition profiles compared to the corresponding WT virus, regardless of the NA subtype, N1 or N8 (Table S3). Notably, the introduction of the T156A substitution in clade 2.3.4 H5 viruses resulted in high cross-reactivity to both clade 2.3.4- and 2.3.4.4-specific antisera, while the A156T substitution in clade 2.3.4.4 H5 viruses resulted in complete loss of cross-reactivity to both anti-sera (Table S3).

**Figure 6.**
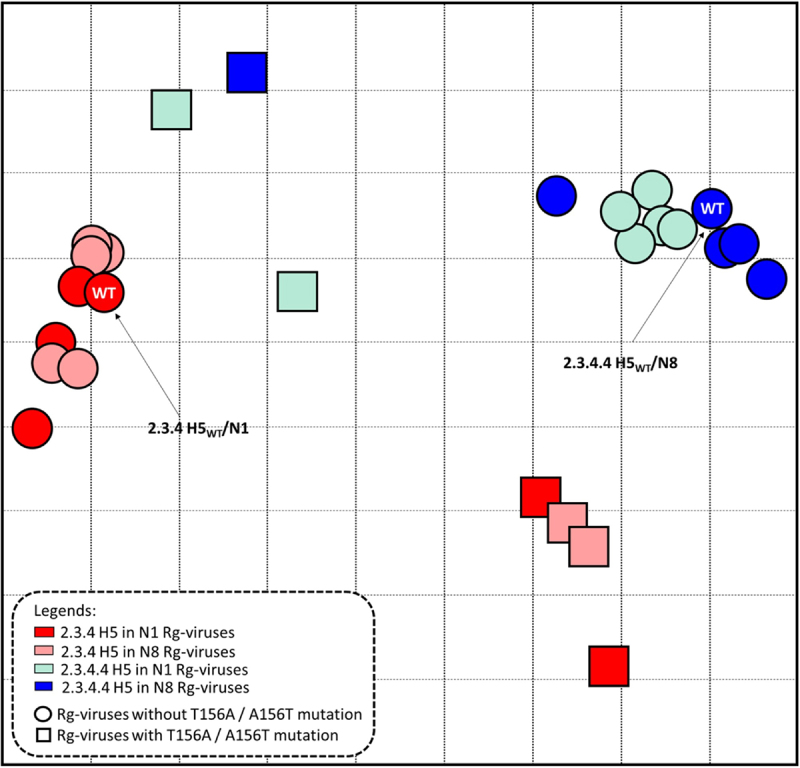
**Antigenic drift of 2.3.4 and 2.3.4.4 H5 *Rg* viruses demonstrated recognition by specific 2.3.4 and 2.3.4.4 H5**_**WT**_**anti-sera**. Antigenic differences between the groups of *Rg* viruses, including both the WT 2.3.4 and 2.3.4.4 H5 and single- or multiple-mutant viruses with N1 or N8 are shown through an antigenic map generated using hemagglutination inhibition (HI) titers derived from recognition of specific anti-sera for the WT 2.3.4 H5N1 and 2.3.4.4 H5N8 HA proteins (Table S3). The antigenic cartography map was constructed using the constructed HI matrices with the subsets of viruses against the two anti-sera normalized by low-rank matrix completion. Using multidimensional scaling in the R program, the viruses were projected into a 2D map wherein each grid line (horizontal and vertical) in the map represents a distance of one antigenic unit (AU), corresponding to a 2-fold difference in HI titers. The Euclidean distances corresponding to the antigenic distance differences among the viruses were calculated and are presented in Table S4.

To characterize the antigenic evolution of the *Rg* viruses, a two-dimensional antigenic cartography map was generated [35,44,45]. Antigenic cartography maps aid in visualizing the antigenic distances between *Rg* viruses based on their quantified HI serum reactions [45]. The cartography map revealed distinct viral clusters among the subsets of *Rg* viruses (Figure 6). The map shows that 2.3.4 H5 and 2.3.4.4 H5 *Rg* viruses without the T156A and A156T mutations, respectively, clustered closely with the corresponding WT viruses. Interestingly, introduction of T156A led to an antigenically distinct cluster of 2.3.4 H5 *Rg* viruses (8.759–10.770 distance difference from the 2.3.4 H5_WT_/N1) (Figure 6 and Table S4). Likewise, 2.3.4.4 H5 *Rg* viruses with A156T moved closer to the 2.3.4 H5 cluster (8.000–9.055 distance difference from 2.3.4.4 H5_WT_/N8) but were still distant from the 2.3.4 H5_WT_/N1 cluster (3.585–4.693 distance differences). The *Rg* viruses with a substitution at residue 156 alone or in combination with the other mutations were located between the clusters of clade 2.3.4 and 2.3.4.4 H5 Rg viruses without the T156A/A156T mutation (Figure 6). The dramatic changes in the cross-reactivity of *Rg* viruses with substitution at residue 156 highlight the critical role of the T156A residue in an antigenic drift.

## Discussion

From 2006 to 2010, a decline in the viral population was observed among the circulating long-stable 2.3.4 H5N1 [2,9,18,46,47], followed by the consequential emergence and spread of H5Nx viruses in 2008 [9,18,41,48]. These changes can possibly be attributed to the naturally extensive genetic reassortment activity of HPAI H5 viruses; to several contributing factors, such as migratory fowl, flyways, and poultry farming practices, including the distribution of vaccines such as Re-5 (generated from clade 2.3.4 H5N1) in 2008; or a combination of the above reasons [9,18,46,47,49]. From the sequence of events, China’s forced mass vaccinations may have exerted selective pressure driving the emergence of escape mutations that could have led to this antigenic drift [9,47]. Here, we demonstrated that the T156A-S123P-D183N-S223 R combined mutation in the H5 gene of clade 2.3.4 viruses is responsible for the altered NA selection preference of these viruses. Additionally, in the developed NA selection assay, host factors rather than IGCs primarily contributed to the selection process. The combination of the four AA substitutions in H5 in 2.3.4 viruses containing N8 restored their replicative ability and improved the HA and NA activities, as revealed by the similarity of the activities of its glycoproteins to those of the 2.3.4.4 H5_WT_/N8 virus. Under natural selection, 2.3.4 H5_T156A-S123P-D183N-S223 R_/N1- and Nx-like viruses may have co-circulated and persisted over time. Interestingly, one of the first H5N5 viruses reported [12] was identified as the prototype of the 2.3.4 H5 virus after the T156A mutation occurred in the presence of S123P-D183N-S223 R substitutions in 2008 [41]. This finding supports our hypothesis that 2.3.4 H5 viruses with residues T156A-S123P-D183N-S223 R persisted over only a short period and subsequently adopted a compensatory mechanism of NA alteration.

The failed attempts to regenerate *Rg* viruses in 2.3.4.4 with reverse substitutions in N8 (2.3.4.4 H5_A156T-P123S-N183D-R223S_/N8) indicate that the current 2.3.4.4 H5N8 viruses have markedly compensated for the changes in their innate functionality, affecting overall viral fitness. This finding also suggests that the irreversible change in H5 in the current 2.3.4.4 H5N8 background can dramatically alter the antigenicity of both 2.3.4 and 2.3.4.4 H5 viruses. The lack of cross-reactivity of 2.3.4.4 H5 with the A156T mutation to clade 2.3.4 or 2.3.4.4 anti-sera also indicates that the other mutation/s in the current 2.3.4.4 HA, which has not been studied here, may influence the antigenicity of the epitope for antibody binding.

**Figure 7.**
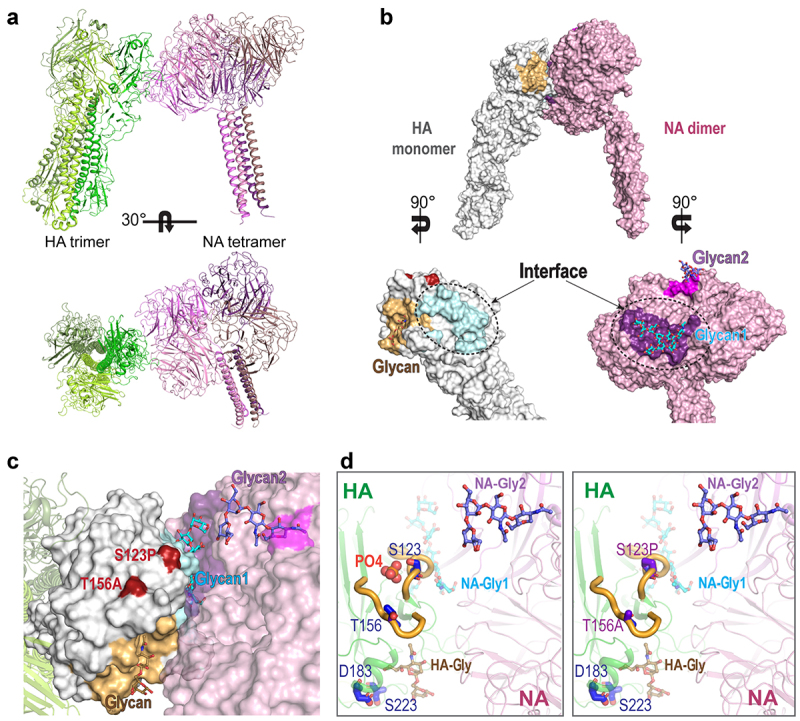
**Structural modeling of 2.3.4 Rg viruses with WT H5 and HA with combination mutations**. (A) Docking model of the influenza a HA trimer and NA tetramer. (B) Predicted contact regions (dashed line) for docked structures identified in HA (protein data bank (PDB) id: 4bh0) (cyan) and NA (PDB id: 6crd) (purple) using the ZDOCK server. The glycan-binding sites are colored bright orange (HA), magenta, and purple (both in NA). The contact region in HA is colored pale cyan. The identified NA contact site also overlaps with a sialic acid-binding site. Among the four identified mutations in 2.3.4 H5, S123P and T156A (red) are located near the contact interface. (C) the HA-NA contact interface in the docking model and the site of glycan binding are presented in detail, with glycans shown as sticks. (D) Details regarding the four mutation sites in 2.3.4 H5 identified in this study, which are shown as blue sticks in their respective loop (thick yellow) locations. A phosphate molecule (orange spheres) is depicted between the loops near residues S123 and T156 in the WT structure of HA. AA substitutions of S123 and T156 to pro and ala, respectively, leading to the loss of phosphate molecules that may have elicited conformational changes affecting the stability and flexibility of these loops and the overall interaction with NA. All images were generated using PyMOL.

Structurally, *N*-glycosylation (-N-X-T/S-) plays a large role in host immune response escape [50], which promotes influenza virus antigenic drift and potential antigenic shifts (according to this study). Thus, the loss of *N*-linked glycosylation at N154 due to the T156A substitution potentially affected the HA-NA interaction, resulting in either decreased N1 selection or enhanced preference of other Nx proteins for cleavage of the sialic acid bound to HA. As both glycoproteins recognize the same sialic acid, there is a functional balance between the complex interaction of HA in receptor binding and NA receptor destruction [19,20]. The structural changes due to the lack of a phosphate molecule interacting with S123 and T156 may affect the overall stability and flexibility of the structural loops of HA at the NA surface interaction site (Figure 7). The conformational changes due to the D183N and S223 R substitutions may have an impact on the virus’s receptor-binding activity. Hence, the S123P and T156A substitutions may have directly affected the balance of functional activity between the two glycoproteins, making HA more flexible and more readily accessible for interaction with its own or another NA protein (Figure 7(a-d)).

Guo et al. revealed that 2.3.4.4 H5Nx viruses have increased binding ability to fucosylated receptors, 3’SLeX, which are considered as “poultry-specific” adaptation receptors [41]. Hiono et al. also mentioned that these 3’SLeX−type receptors can be readily found on the intestinal epithelial cells of different avian species [51] which may explain the strong host-specific selection observed from N8 and other Nx of 2.3.4.4 H5 gene when passaged in chicken embryonated eggs compared to MDCK cells. Although previously, Guo et al [41]. evaluated and showed K218Q-S223 R combination’s impact on the altered-binding specificity of 2.3.4.4 H5 viruses using fucosylated glycans, in our study, mutation combination in H5 was not recovered in this 2.3.4 H5 virus. The use of prototype viruses and fucosylated glycans could better aid in understanding the role of other conserved substitutions, such as K82 R, K218Q, and N240 H in preferential NA selection. Although this study evaluated the impact of functional balance on influenza glycoproteins, the complexity of the interaction mechanism between the HA-NA protein structure remains elusive; hence, we recommend further study and analysis.

Overall, our study demonstrated that the substitutions in the H5 protein of clade 2.3.4 viruses may have resulted in the preferential selection of other Nx genes and the emergence of novel 2.3.4.4 viruses. Evidently, any modification to the HA as well as the NA gene would likely result in loss of fitness. Several studies have observed compensatory mutations in HA and NA genes of novel reassortment viruses in other subtypes, potentially improving their balance between HA and NA [52–54], although whether the mutations drove or triggered the antigenic shift in the subtypes is inconclusive. As a compensatory mechanism, the accumulated mutations in HA of clade 2.3.4 H5 viruses alter their preference for N1 to that for other Nx genes. More specifically, we were able to provide evidence that key mutations, particularly T156A, led to the 2.3.4 H5 antigenic shift and the emergence of 2.3.4.4 H5Nx viruses in the natural environment. With the increasing potential threat of avian influenza outbreaks, our study can contribute to the understanding of the potential evolutionary mechanism of HPAI viruses and further emphasizes the crucial role of intensive surveillance for 2.3.4.4 H5Nx viruses in monitoring the key molecular changes that may cause the next novel HPAI emergence.

## Supplementary Material

Supplemental Material

## Data Availability

Data available within the article or its supplementary materials.
