# Supplementary material for: Multiple HA substitutions in highly pathogenic avian influenza H5Nx viruses contributed to the change in the NA subtype preference
Source: Virulence. 2022 Jun 5;13(1):990–1004. doi: 10.1080/21505594.2022.2082672 (PMC9176248; doi:10.1080/21505594.2022.2082672)
Supplement: Supplemental Material [file KVIR_A_2082672_SM1328.pdf]

Supplementary Material for Publication

Table of Contents:

Supplementary Figure S1	Schematic diagram of the NA selection assay.
Supplementary Figure S2	Glycoprotein activities of 2.3.4.4 H5 WT viruses and those with combination HA gene substitutions.
Supplementary Figure S3	Glycoprotein activities of 2.3.4 and 2.3.4.4 H5 with single mutants.
Supplementary Figure S4	HA binding activities of 2.3.4 and 2.3.4.4 viruses determined using α2,6 biotinylated glycans.
Supplementary Figure S5	Comparison of NA enzymatic activity among the WT and selected 2.3.4 Rg viruses in N1 and N8.
Supplementary Table S1	Sequence variation analysis of clade 2.3.4 and 2.3.4.4 H5 viruses from 2005-2013.
Supplementary Table S2	HA and NA parameters for selected Rg-viruses with WT or mutated residues in their H5 gene for clades 2.3.4 and 2.3.4.4 carrying N1 and N8
Supplementary Table S3	Haemagglutination inhibition titres of all Rg viruses tested against 2.3.4 and 2.3.4.4 anti-sera
Supplementary Table S4.	Estimated antigenic unit distance differences of each group based on their calculated group mean Euclidean distance.
Supplementary Table S5	NA selection assay primer list

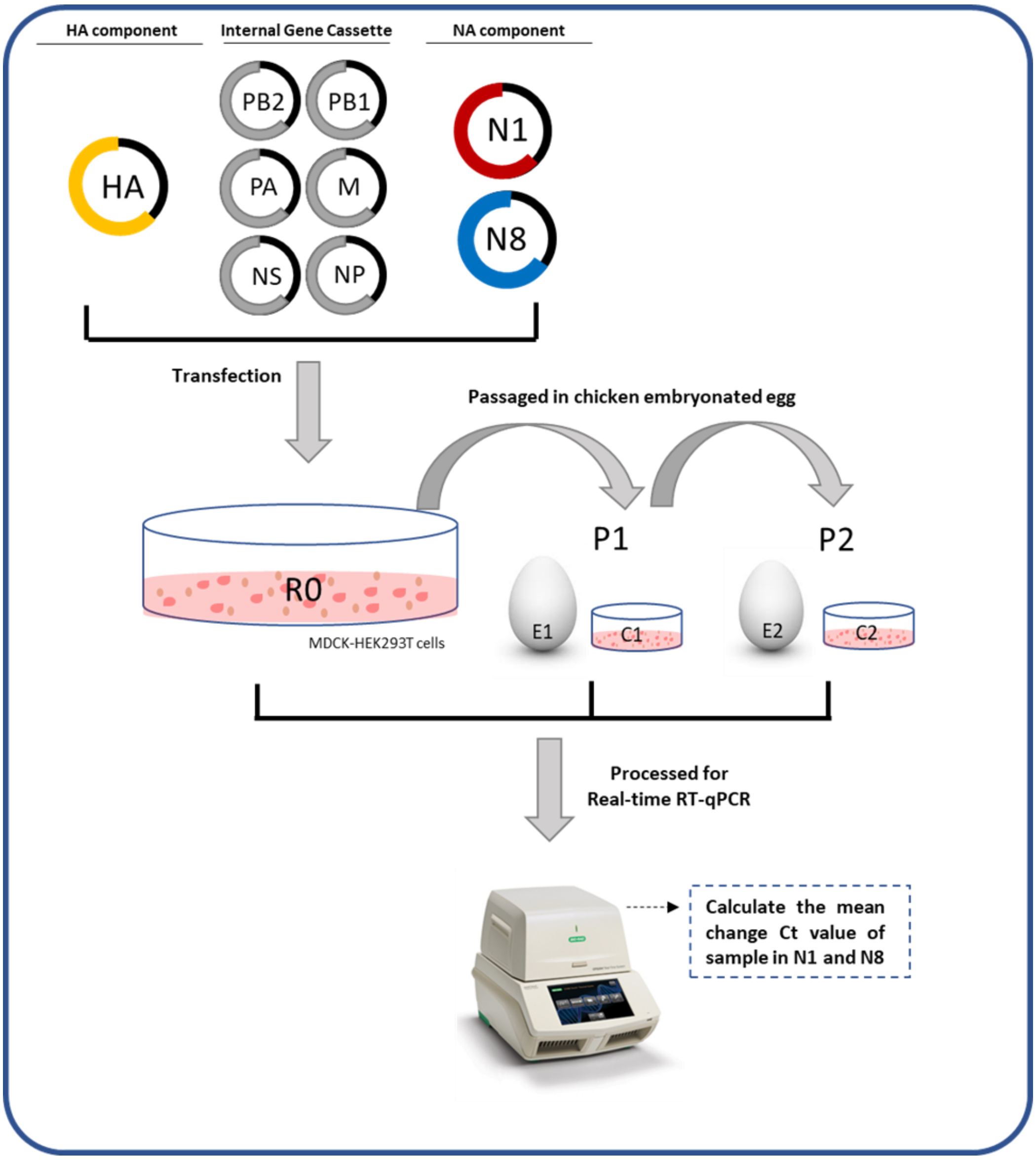

Figure S1. Schematic diagram of the NA selection assay. The in vitro selection assay of the selection preference for NA utilizes reverse genetics. In the process of generating the Rg virus, a set of internal gene cassettes of the same viral origin (human H1N1 (hH1N1), clade 2.2 and clade 2.3.4.4 IGCs), an HA gene (WT or with mutation/s) and two selected NA genes of N1 and N8 were combined and transfected into a mixture of Madin-Darby canine kidney (MDCK) cells and human embryonic kidney (HEK) 293T cells. Rescue supernatants were harvested 5 days after transfection and passaged twice in 10-day-old embryonated chicken eggs and/or MDCK cells. Forty-eight hours post inoculation, the allantoic fluid and/or MDCK supernatant were harvested and treated with DNase during RNA extraction with a Qiagen RNA Extraction Kit. Samples from the rescue supernatant (R0), passage one (E1/C1), and passage two (E2/C2) were analysed by real-time quantitative (q)PCR. The change in mean Ct values for each sample processed was recorded and presented in a graph using GraphPad Prism 9.1. The mean ΔCt values represent the preference for the representative NA subtype, N1 or N8.

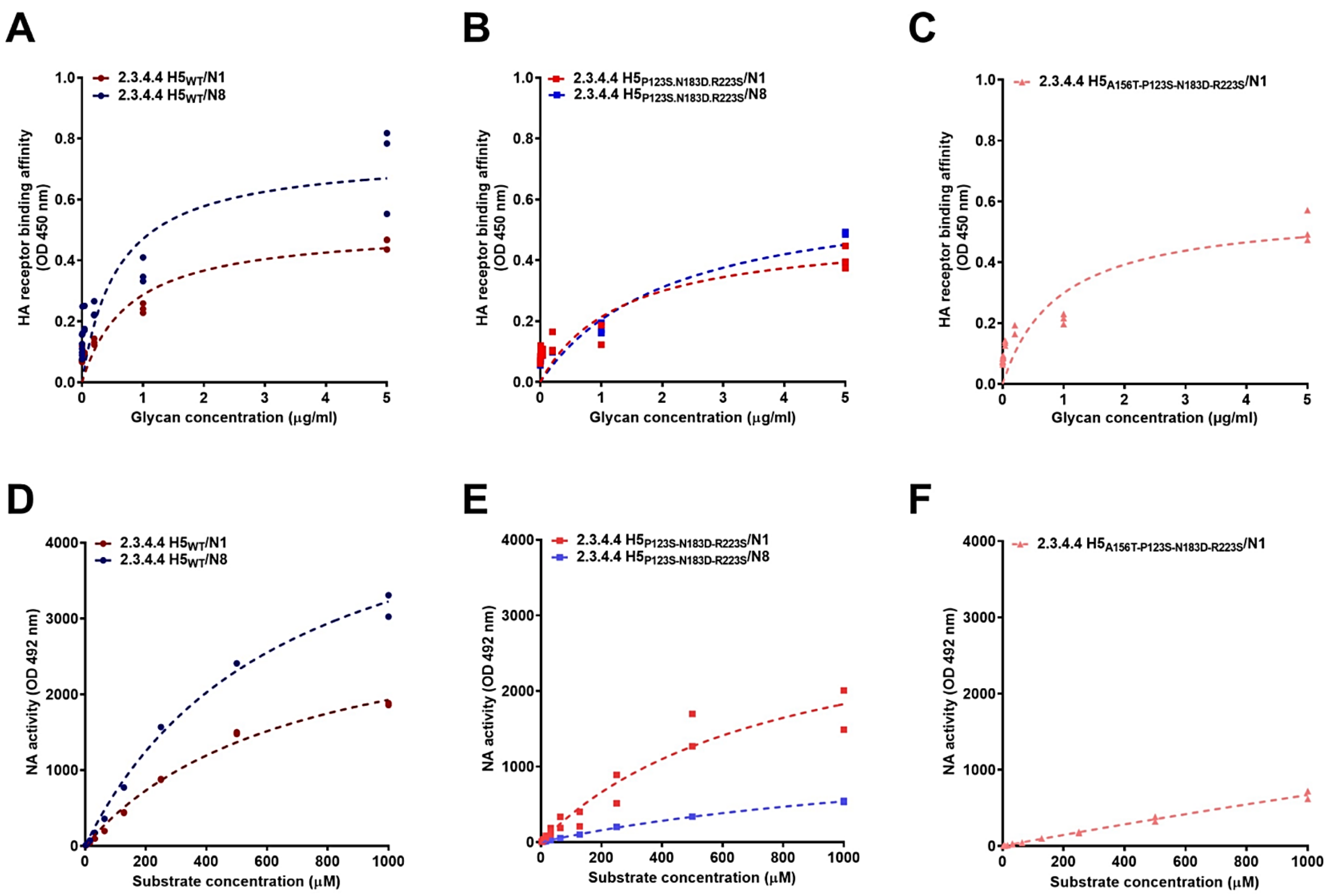

Figure S2. Glycoprotein activities of 2.3.4.4 H5 WT viruses and those with combination HA gene substitutions. The 2.3.4.4-representative Rg viruses were evaluated according to their respective HA and NA activities. The HA receptor binding affinity of 2.3.4.4 H5$_{\text{WT}}$, 2.3.4.4 H5$_{\text{P123S-N183D-R223S}}$, and 2.3.4.4 H5$_{\text{A156T-P123S-N183D-R223S}}$ with N1 or N8 was measured using α2,3 biotinylated glycans via a solid-phase-based enzyme-linked assay read at OD 450 nm. A comparison of HA receptor binding affinity between 2.3.4.4 H5 *Rg* viruses with N1 and N8 (A, B, and C) is shown. The NA activity of 2.3.4.4 H5$_{\text{WT}}$, 2.3.4.4 H5$_{\text{P123S-N183D-R223S}}$, and 2.3.4.4 H5$_{\text{A156T-P123S-N183D-R223S}}$ with N1 or N8 was also determined through a chemiluminescence assay read at OD 492 nm. A comparison of NA activity between 2.3.4.4 H5 *Rg* viruses with N1 and N8 is also shown (D, E and F). The results presented are the mean values of minimum of 2 repetitions of the assays.

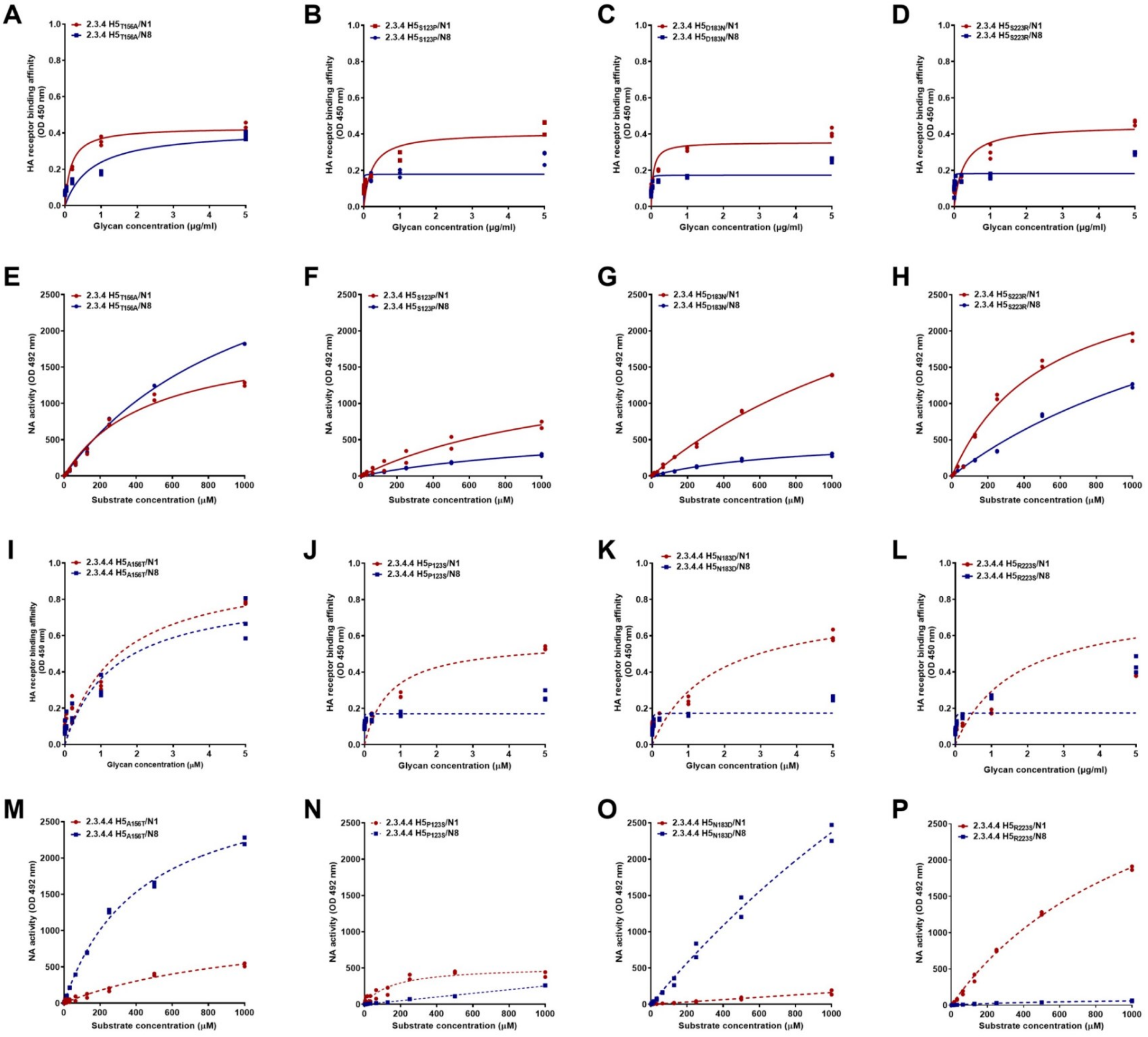

Figure S3. Glycoprotein activities of 2.3.4 and 2.3.4.4 H5 with single mutants. HA receptor binding assays were performed using α2,3 biotinylated glycans among 2.3.4 H5 (A, B, C, and D) and 2.3.4.4. H5 (I, J, K, and L) with single mutations. NA activity was also determined through the NA-Star chemiluminescence assay, and the signal was read at OD 492 nm (E, F, G, H, M, N, O, and P). The results presented are the mean values of minimum 3 repetitions of the performed assays.

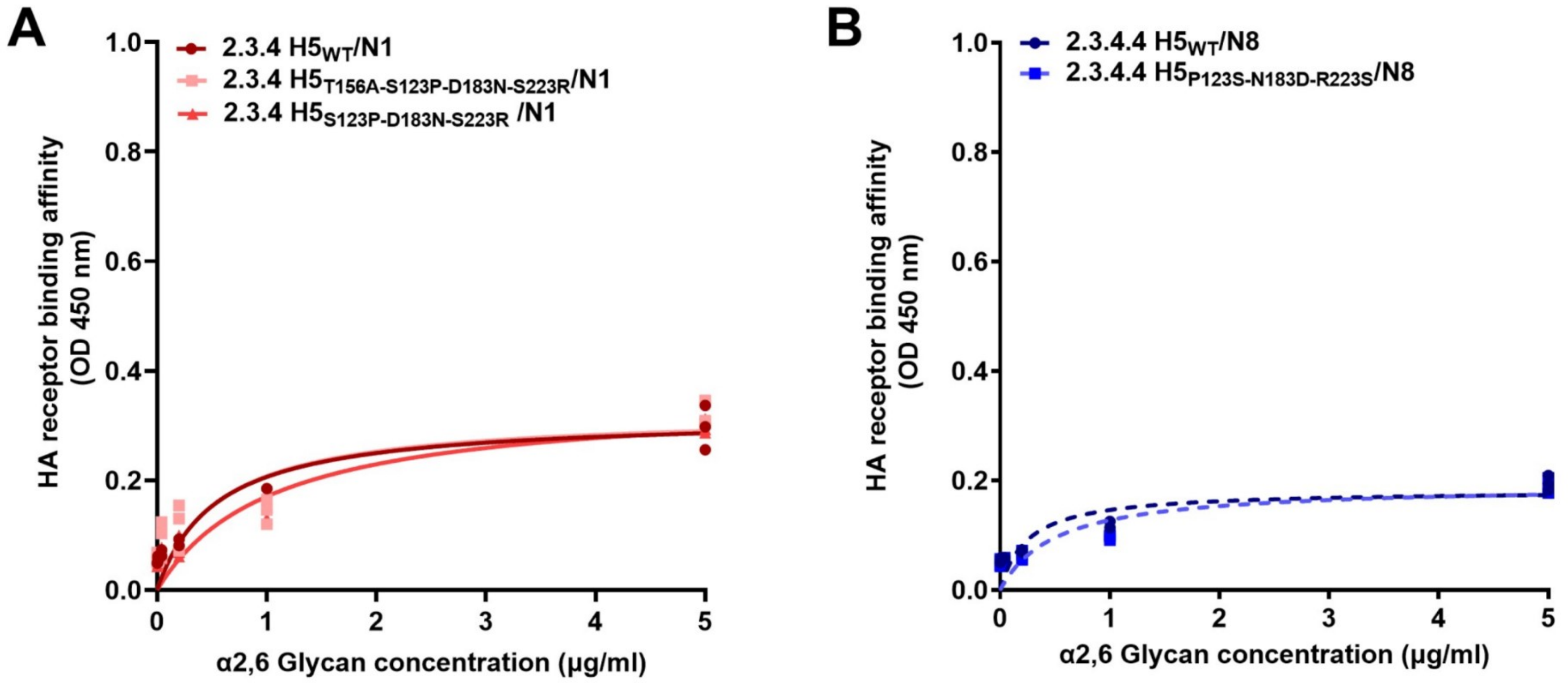

Figure S4. HA binding activities of 2.3.4 and 2.3.4.4 viruses determined using α2,6 biotinylated glycans. The HA receptor binding affinities towards α2,6 receptors for 2.3.4 H5 viruses with N1 and 2.3.4.4 H5, both WT and with 3-4 combined amino acid substitutions, were determined. The HA binding affinities using α2,6 biotinylated glycans were evaluated using a solid-phase enzyme-linked receptor-binding assay. The results presented are the mean values of minimum 3 repetitions of the assays.

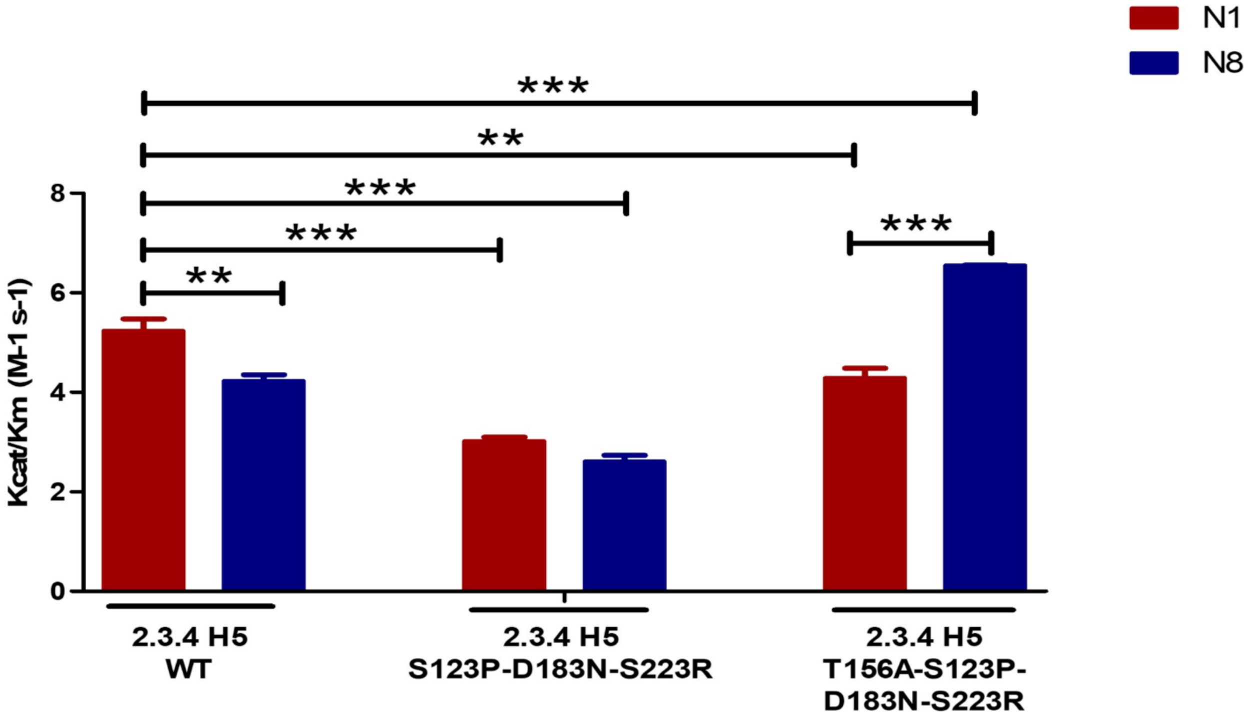

Figure S5. Comparison of NA enzymatic activity among the WT and selected 2.3.4 *Rg* viruses in N1 and N8. Determination of the NA kinetic constants Km, Vmax and Kcat of 2.3.4 $H5_{WT}$, 2.3.4 $H5_{S123P\text{-}D183N\text{-}S223R}$, and 2.3.4 $H5_{T156A\text{-}S123P\text{-}D183N\text{-}S223R}$ with N1 or N8 by the use of the enzyme kinetic assay using the fluorogenic substrate 2'-(4-methylumbelliferyl)-α-D-N-acetylneuraminic acid (MUNANA) (data obtained from Table S2). The results presented are the mean Kcat/Km values of 3 repetitive assay determinations. The error bars represent standard errors. Significant results according to the calculated *P*-values (**, $P \leq 0.05$; ***, $P \leq 0.005$) are indicate

Supplementary Table S1. Sequence variation analysis of clade 2.3.4 and 2.3.4.4 H5 viruses from 2005-2013.

		Polymorphisms in database (% frequency)															
Year of sample isolation		K82R		S123P		T156A		D183N		K218Q		S223R		N240H		n	Total number of sequences analysed
2005	H5N1	Lys	100.00	Ser	100.00	Thr	100.00	Asp	100.00	Lys	100.00	Ser	100.00	Asn	100.00	38	38
2006	H5N1	Lys	100.00	Ser	86.67	Thr	97.78	Asp	91.11	Lys	100.00	Ser	97.78	Asn	100.00	45	45
				Pro	8.89	Ile	2.22	Asn	8.89			Xaa	2.22				
				Phe	4.44												
2007	H5N1	Lys	100.00	Ser	100.00	Thr	98.25	Asp	98.25	Lys	100.00	Ser	100.00	Asn	100.00	57	57
						Ala	1.75	Asn	1.75								
2008	H5N1	Lys	100.00	Ser	90.91	Thr	100.00	Asp	100.00	Lys	81.82	Ser	100.00	Asn	90.91	11	16
										Arg	9.09			Tyr	9.09		
				Pro	9.09					Gln	9.09						
	H5Nx	Arg	100.00	Pro	100.00	Thr	20.00	Asn	100.00	Lys	100.00	Arg	100.00	His	100.00	5	
						Ala	80.00										
2009	H5N1	Lys	100.00	Ser	13.33	Thr	100.00	Asp	66.67	Lys	100.00	Ser	100.00	Asn	100.00	15	19
				Pro	86.67			Asn	33.33								
	H5Nx	Arg	100.00	Pro	100.00	Thr	25.00	Asn	75.00	Gln	100.00	Arg	100.00	His	100.00	4	
						Ala	75.00	Asp	25.00								
2010	H5N1	Lys	100.00	Ser	7.14	Thr	92.86	Asp	14.29	Lys	100.00	Ser	92.86	Asn	100.00	14	20
				Pro	92.86	Ala	7.14	Asn	85.71			Gly	7.14				
	H5Nx	Arg	100.00	Pro	100.00	Ala	100.00	Asn	100.00	Gln	100.00	Arg	100.00	His	100.00	6	
2011	H5N1	Lys	100.00	Pro	100.00	Thr	100.00	Asn	100.00	Lys	100.00	Ser	100.00	Asn	100.00	6	12
	H5Nx	Arg	100.00	Pro	100.00	Ala	100.00	Asn	100.00	Gln	100.00	Arg	100.00	His	100.00	6	

2012	H5N1	Lys	100.00	Pro	100.00	Thr	100.00	Asn	100.00	Lys	100.00	Ser	100.00	Asn	100.00	2	9
	H5Nx	Arg	100.00	Pro	100.00	Ala	100.00	Asn	100.00	Gln	100.00	Arg	100.00	His	100.00	7	
2013	H5N1	Lys	100.00	Ser	100.00	Thr	100.00	Asp	100.00	Thr	100.00	Ser	100.00	Asn	100.00	1	42
	H5Nx	Arg	100.00	Pro	65.85	Ala	100.00	Asn	100.00	Gln	100.00	Arg	100.00	His	100.00	41	
				Leu	9.76												
				Thr	24.39												

Supplementary Table S2. HA and NA parameters for selected Rg-viruses with WT or mutated residues in their H5 gene for clades 2.3.4 and 2.3.4.4 carrying N1 and N8

	Haemagglutinin binding parameters[A]	Neuraminidase enzyme kinetics parameters[B]			
**Influenza Rg viruses**	**Kd (µM)**	**Vmax (µM/min)**	**Km (µM)**	**Kcat (s-1)**	**Kcat/Km (M-1 s-1)**
2.3.4 $H5_{WT}$/N1	0.665 ± 0.16	0.5373 ± 0.03	16.69 ± 0.06	8.74E-05	5.24 ± 0.23
2.3.4 $H5_{WT}$/N8	1.871 ± 1.75	0.8647 ± 0.07	33.23 ± 0.23	1.41E-04	4.23 ± 0.12
2.3.4 $H5_{S123P\text{-}D183N\text{-}S223R}$/N1	2.517 ± 1.29	0.4556 ± 0.04	24.53 ± 0.09	7.41E-05	3.02 ± 0.08
2.3.4 $H5_{S123P\text{-}D183N\text{-}S223R}$/N8	3.149 ± 1.73	0.1807 ± 0.16	40.95 ± 0.98	1.07E-04	2.61 ± 0.12
2.3.4 $H5_{T156A\text{-}S123P\text{-}D183N\text{-}S223R}$/N1	0.468 ± 0.18	0.6168 ± 0.07	17.95 ± 1.81	8.91E-05	4.95 ± 0.27
2.3.4 $H5_{T156A\text{-}S123P\text{-}D183N\text{-}S223R}$/N8	0.529 ± 0.28	0.5349 ± 0.02	13.29 ± 0.04	8.70E-05	6.55 ± 0.01
2.3.4.4 $H5_{WT}$/N8	0.591 ± 0.29	0.9529 ± 0.01	109.07 ± 0.17	5.16E-04	4.73 ± 0.02
2.3.4.4 $H5_{WT}$/N1	0.770 ± 0.25	0.4369 ± 0.01	26.52 ± 0.08	2.58E-04	9.73 ± 0.01
2.3.4.4 $H5_{P123S\text{-}N183D\text{-}R223S}$/N8	2.149 ± 1.19	0.2 ± 0.01	50.12 ± 1.31	1.18E-04	2.36 ± 0.21
2.3.4.4 $H5_{P123S\text{-}N183D\text{-}R223S}$/N1	1.329 ± 0.88	0.4537 ± 0.07	20.71 ± 0.01	2.68E-04	12.95 ± 0.08
2.3.4.4 $H5_{A156T\text{-}P123S\text{-}N183D\text{-}R223S}$/N8	-	-	-	-	-
2.3.4.4 $H5_{A156T\text{-}P123S\text{-}N183D\text{-}R223S}$/N1	0.917 ± 0.46	0.3949 ± 0.02	45.04 ± 0.04	2.33E-04	5.17 ± 0.92

[A]Determination of the Kd value were fitting into a non-linear regression analysis using one-site specific in a formula Y=Bmax*X/(Kd+X) + NS*X + Background binding in GraphPad 9.1 software. Bmax represents the maximum specific binding; NS value as the slope of nonspecific binding in Y units divided by X units; while the amount of nonspecific binding is measured without the added glycans were presented as background

[B]Determination of the neuraminidase kinetic constants Km, Vmax and Kcat by using the fluorogenic substrate 2′-(4-methylumbelliferyl)-α-D-N-acetylneuraminic acid (MUNANA), as presented in the data table.

Note: The results presented are the mean values of three experimental determinations with three replicates per determination.

‘-’ means not determined.

Supplementary Table S3. Haemagglutination inhibition titres of all Rg viruses tested against 2.3.4 and 2.3.4.4 anti-sera

Rg viruses	2.3.4 Anti-sera	2.3.4.4 Anti-sera
	(HI titre mean values ± SD)	(HI titre mean values ± SD)
2.3.4 $H5_{WT}$/N1	160 ± 0	13 ± 23
2.3.4 $H5_{T156A}$/N1	2560 ± 0	13653 ± 5912
2.3.4 $H5_{S123P}$/N1	160 ± 0	<10
2.3.4 $H5_{D183N}$/N1	427 ± 185	<10
2.3.4 $H5_{S223R}$/N1	1067 ± 370	<10
2.3.4 $H5_{S123P-D183N-S223R}$/N1	373 ± 244	<10
2.3.4 $H5_{T156A-S123P-D183N-S223R}$/N1	427 ± 185	3413 ± 1478
2.3.4 $H5_{WT}$/N8	107 ± 46	<10
2.3.4 $H5_{T156A}$/N8	640 ± 0	6827 ± 2956
2.3.4 $H5_{S123P}$/N8	853 ± 370	27+23
2.3.4 $H5_{D183N}$/N8	427 ± 185	<10
2.3.4 $H5_{S223R}$/N8	107 ± 46	<10
2.3.4 $H5_{S123P-D183N-S223R}$/N8	107 ± 46	<10
2.3.4 $H5_{T156A-S123P-D183N-S223R}$/N8	533 ± 185	5120 ± 0
2.3.4.4 $H5_{WT}$/N1	7 ± 12	3413 ± 1478
2.3.4.4 $H5_{A156T}$/N1	13 ± 23	13 ± 12
2.3.4.4 $H5_{P123S}$/N1	<10	5120 ± 0
2.3.4.4 $H5_{N183D}$/N1	<10	4267 ± 1478
2.3.4.4 $H5_{R223S}$/N1	<10	2560 ± 2217
2.3.4.4 $H5_{P123S-N183D-R223S}$/N1	13 ± 23	3413 ± 1478
2.3.4.4 $H5_{A156T-P123S-N183D-R223S}$/N1	80 ± 0	107 ± 46
2.3.4.4 $H5_{WT}$/N8	7 ± 12	6827 ± 2956
2.3.4.4 $H5_{A156T}$/N8	7 ± 12	27 ± 46
2.3.4.4 $H5_{P123S}$/N8	<10	1280 ± 0
2.3.4.4 $H5_{N183D}$/N8	<10	10240 ± 0
2.3.4.4 $H5_{R223S}$/N8	<10	10240 ± 0
2.3.4.4 $H5_{P123S-N183D-R223S}$/N8	13 ± 23	17067 ± 5912
2.3.4.4 $H5_{A156T-P123S-N183D-R223S}$/N8	n/a	n/a

* The results are the Log_2 mean values of three experimental determinations with three replicates per determination;
HI titres of viruses observed <10 represent samples with cross-reactivity below the detection limit.
Abbrev: SD: Standard deviation; WT: wild type; n/a: not applicable;

Supplementary Table S4. Estimated antigenic unit distance differences of each group based on their calculated group mean Euclidean distance.

AU differences of each Rg-viruses	2.3.4 $H5_{WT}$/N1	2.3.4 $H5_{WT}$/N1	2.3.4 $H5_{T156A}$/N1	2.3.4 $H5_{S123P}$/N1	2.3.4 $H5_{D183N}$/N1	2.3.4 $H5_{S223R}$/N1	2.3.4 $H5_{S123P-D183N-S223R}$/N1	2.3.4 $H5_{T156A-S123P-D183N-S223R}$/N1	2.3.4.4 $H5_{WT}$/N1	2.3.4.4 $H5_{A156T}$/N1	2.3.4.4 $H5_{P123S}$/N1	2.3.4.4 $H5_{N183D}$/N1	2.3.4.4 $H5_{R223S}$/N1	2.3.4.4 $H5_{P123S-N183D-R223S}$/N1	2.3.4.4 $H5_{A156T-P123S-N183D-R223S}$/N1	2.3.4 $H5_{WT}$/N8	2.3.4 $H5_{T156A}$/N8	2.3.4 $H5_{S123P}$/N8	2.3.4 $H5_{D183N}$/N8	2.3.4 $H5_{S223R}$/N8	2.3.4 $H5_{S123P-D183N-S223R}$/N8	2.3.4 $H5_{T156A-S123P-D183N-S223R}$/N8	2.3.4.4 $H5_{WT}$/N8	2.3.4.4 $H5_{A156T}$/N8	2.3.4.4 $H5_{P123S}$/N8	2.3.4.4 $H5_{N183D}$/N8	2.3.4.4 $H5_{R223S}$/N8
2.3.4 $H5_{WT}$/N1	0.000	10.770	0.415	1.475	2.768	1.291	8.124	9.221	3.585	9.471	9.233	8.575	8.767	3.162	0.717	9.220	1.415	1.475	0.717	0.717	8.759	10.101	4.693	7.705	10.386	10.386	10.927
2.3.4 $H5_{T156A}$/N1	10.770	0.000	11.157	10.731	10.491	10.779	3.268	8.815	12.551	8.124	8.174	8.357	7.844	8.602	11.380	2.236	10.329	10.731	11.380	11.380	2.669	8.643	12.438	8.698	8.011	8.011	7.592
2.3.4 $H5_{S123P}$/N1	0.415	11.157	0.000	1.415	2.737	1.222	8.533	9.583	3.609	9.849	9.609	8.944	9.147	3.558	0.585	9.625	1.475	1.415	0.585	0.585	9.166	10.472	4.798	8.062	10.770	10.770	11.320
2.3.4 $H5_{D183N}$/N1	1.475	10.731	1.415	0.000	1.322	0.193	8.415	10.335	5.017	10.503	10.279	9.660	9.788	4.183	2.000	9.433	0.415	0.000	2.000	2.000	9.006	11.164	6.165	8.850	11.372	11.372	11.844
2.3.4 $H5_{S223R}$/N1	2.768	10.491	2.737	1.322	0.000	1.515	8.518	11.155	6.336	11.242	11.033	10.459	10.525	5.062	3.322	9.444	1.385	1.322	3.322	3.322	9.055	11.927	7.457	9.715	12.058	12.058	12.460
2.3.4 $H5_{S123P-D183N-S223R}$/N1	1.291	10.779	1.222	0.193	1.515	0.000	8.417	10.224	4.825	10.405	10.179	9.554	9.691	4.074	1.807	9.447	0.457	0.193	1.807	1.807	9.015	11.062	5.977	8.733	11.282	11.282	11.764
2.3.4 $H5_{T156A-S123P-D183N-S223R}$/N1	8.124	3.268	8.533	8.415	8.518	8.417	0.000	6.000	9.434	5.447	5.425	5.431	5.000	5.553	8.649	1.159	8.000	8.415	8.649	8.649	0.668	6.083	9.220	5.597	5.642	5.642	5.513
2.3.4.4 $H5_{WT}$/N1	9.221	8.815	9.583	10.335	11.155	10.224	6.000	0.000	8.062	0.827	0.668	0.717	1.000	6.152	9.317	6.660	10.000	10.335	9.317	9.317	6.349	1.000	7.000	1.531	1.689	1.689	2.528
2.3.4.4 $H5_{A156T}$/N1	3.585	12.551	3.609	5.017	6.336	4.825	9.434	8.062	0.000	8.595	8.332	7.596	8.000	3.960	3.029	10.592	5.000	5.017	3.029	3.029	10.101	9.055	1.414	6.598	9.594	9.594	10.322
2.3.4.4 $H5_{P123S}$/N1	9.471	8.124	9.849	10.503	11.242	10.405	5.447	0.827	8.595	0.000	0.263	1.000	0.717	6.340	9.626	6.014	10.150	10.503	9.626	9.626	5.737	0.717	7.607	2.000	1.000	1.000	1.786
2.3.4.4 $H5_{N183D}$/N1	9.233	8.174	9.609	10.279	11.033	10.179	5.425	0.668	8.332	0.263	0.000	0.737	0.525	6.109	9.381	6.038	9.929	10.279	9.381	9.381	5.743	0.896	7.345	1.737	1.263	1.263	2.043
2.3.4.4 $H5_{R223S}$/N1	8.575	8.357	8.944	9.660	10.459	9.554	5.431	0.717	7.596	1.000	0.737	0.000	0.587	5.479	8.698	6.165	9.320	9.660	8.698	8.698	5.823	1.531	6.611	1.000	2.000	2.000	2.768
2.3.4.4 $H5_{P123S-N183D-R223S}$/N1	8.767	7.844	9.147	9.788	10.525	9.691	5.000	1.000	8.000	0.717	0.525	0.587	0.000	5.629	8.934	5.674	9.434	9.788	8.934	8.934	5.354	1.414	7.071	1.475	1.638	1.638	2.322
2.3.4.4 $H5_{A156T-P123S-N183D-R223S}$/N1	3.162	8.602	3.558	4.183	5.062	4.074	5.553	6.152	3.960	6.340	6.109	5.479	5.629	0.000	3.440	6.708	3.852	4.183	3.440	3.440	6.220	6.989	4.105	4.675	7.236	7.236	7.765
2.3.4 $H5_{WT}$/N8	0.717	11.380	0.585	2.000	3.322	1.807	8.649	9.317	3.029	9.626	9.381	8.698	8.934	3.440	0.000	9.763	2.043	2.000	0.000	0.000	9.295	10.230	4.243	7.789	10.567	10.567	11.148
2.3.4 $H5_{T156A}$/N8	9.220	2.236	9.625	9.433	9.444	9.447	1.159	6.660	10.592	6.014	6.038	6.165	5.674	6.708	9.763	0.000	9.019	9.433	9.763	9.763	0.491	6.585	10.362	6.468	6.028	6.028	5.739
2.3.4 $H5_{S123P}$/N8	1.415	10.329	1.475	0.415	1.385	0.457	8.000	10.000	5.000	10.150	9.929	9.320	9.434	3.852	2.043	9.019	0.000	0.415	2.043	2.043	8.591	10.817	6.083	8.526	11.009	11.009	11.470
2.3.4 $H5_{D183N}$/N8	1.475	10.731	1.415	0.000	1.322	0.193	8.415	10.335	5.017	10.503	10.279	9.660	9.788	4.183	2.000	9.433	0.415	0.000	2.000	2.000	9.006	11.164	6.165	8.850	11.372	11.372	11.844
2.3.4 $H5_{S223R}$/N8	0.717	11.380	0.585	2.000	3.322	1.807	8.649	9.317	3.029	9.626	9.381	8.698	8.934	3.440	0.000	9.763	2.043	2.000	0.000	0.000	9.295	10.230	4.243	7.789	10.567	10.567	11.148
2.3.4 $H5_{S123P-D183N-S223R}$/N8	0.717	11.380	0.585	2.000	3.322	1.807	8.649	9.317	3.029	9.626	9.381	8.698	8.934	3.440	0.000	9.763	2.043	2.000	0.000	0.000	9.295	10.230	4.243	7.789	10.567	10.567	11.148
2.3.4 $H5_{T156A-S123P-D183N-S223R}$/N8	8.759	2.669	9.166	9.006	9.055	9.015	0.668	6.349	10.101	5.737	5.743	5.823	5.354	6.220	9.295	0.491	8.591	9.006	9.295	9.295	0.000	6.336	9.874	6.076	5.823	5.823	5.598
2.3.4.4 $H5_{WT}$/N8	10.101	8.643	10.472	11.164	11.927	11.062	6.083	1.000	9.055	0.717	0.896	1.531	1.414	6.989	10.230	6.585	10.817	11.164	10.230	10.230	6.336	0.000	8.000	2.485	0.827	0.827	1.658
2.3.4.4 $H5_{A156T}$/N8	4.693	12.438	4.798	6.165	7.457	5.977	9.220	7.000	1.414	7.607	7.345	6.611	7.071	4.105	4.243	10.362	6.083	6.165	4.243	4.243	9.874	8.000	0.000	5.616	8.605	8.605	9.375
2.3.4.4 $H5_{P123S}$/N8	7.705	8.698	8.062	8.850	9.715	8.733	5.597	1.531	6.598	2.000	1.737	1.000	1.475	4.675	7.789	6.468	8.526	8.850	7.789	7.789	6.076	2.485	5.616	0.000	3.000	3.000	3.760
2.3.4.4 $H5_{N183D}$/N8	10.386	8.011	10.770	11.372	12.058	11.282	5.642	1.689	9.594	1.000	1.263	2.000	1.638	7.236	10.567	6.028	11.009	11.372	10.567	10.567	5.823	0.827	8.605	3.000	0.000	0.000	0.846
2.3.4.4 $H5_{R223S}$/N8	10.386	8.011	10.770	11.372	12.058	11.282	5.642	1.689	9.594	1.000	1.263	2.000	1.638	7.236	10.567	6.028	11.009	11.372	10.567	10.567	5.823	0.827	8.605	3.000	0.000	0.000	0.846
2.3.4.4 $H5_{P123S-N183D-R223S}$/N8	10.927	7.592	11.320	11.844	12.460	11.764	5.513	2.528	10.322	1.786	2.043	2.768	2.322	7.765	11.148	5.739	11.470	11.844	11.148	11.148	5.598	1.658	9.375	3.760	0.846	0.846	0.000

The estimated antigenic distance differences of each viruses were calculated as the mean Euclidean distance (⟦d(p,q)⟧^2=⟦(q_1-p_1)⟧^2+⟦(q_2-p_2)⟧^2), where p and q are defined as the numerical differences of the coordinates of each Rg-virus identified in the Euclidean plane.

Supplementary Table S5. NA selection assay primer list

N1 (Clade 2.2, H5N1)		Sequence	N5 (Clade 2.3.4.4, H5N5)		Sequence
Set 1	F	GAGCGGCTTTGAAATGATCT	Set 1	F	GTCCAGCAAACAAACAAGCA
	R	CCCGCTATATCCTGACCAAT		R	TTGGCCTATTCATTCCGTTC
Set 2	F	CATTAGCGGGCAATTCAT CT	Set 2	F	GGTGCAGACGATGATGCTTA
	R	GGAGTGCTTGTCATTCAG CA		R	TGCTTGTTTGTTTGCTGGAC
Set 3	F	ATTGGCATGGCTCAAATAGG	Set 3	F	GTCCAGCAAACAAACAAGCA
	R	CTTTTGGTTCTCCCAATCCA		R	TTGGCCTATTCATTCCGTTC
Set 4	F	ATGGTGTTTGGATTGGGAGA	Set 4	F	TGGGCAGGAAGGACAATAAG
	R	CTCCCGCTATATCCTGACCA		R	AAAGCATGGAACCAGACAGC
**N1 (Clade 2.3.4, H5N1)**		**Sequence**	**N6 (Clade 2.3.4.4, H5N6)**		**Sequence**
Set 1	F	GGTGGACTGAAACGGACAGT	Set 1	F	TCGAATGCATAGGATGGTCA
	R	GGCCAAGACCAACTCACAGT		R	CCACTGGGCAGATTCCTTTA
Set 2	F	ACATTAGCGGGCAATTCATC	Set 2	F	TGACCAAACCGCTATGTGAA
	R	TGGAGTGCTTGTCATTCAGC		R	CTCGAAATGGGCTCCTATCA
Set 3	F	ATTGGCATGGCTCAAATCGG	Set 3	F	TGAGACCAACCCAACAACAA
	R	CTTTTGGTTCTCCCAATCCA		R	AGAGCAAACATCCTGCATCC
Set 4	F	TGGATTGGGAGAACCAAAAG	Set 4	F	AGGGGTAAAAGGGTTTGCAT
	R	CAGTTCTGGATGCTGGACAA		R	CCCTGACCAATTTTGGTTGT
**N2 (Clade 2.3.4.4, H5N2)**		**Sequence**	**N8 (Clade 2.3.4.4, H5N8)**		**Sequence**
Set 1	F	TGCTATCAATTCGCACTTGG	Set 1	F	ACTGGACGGGAACTAACAGG
	R	AACATGCAACCAAGCCTTTC		R	GCATGAACCGACAAATTGAG
Set 2	F	TGTCATGCAGCCCTGATAAA	Set 2	F	GGGTGCCTACTGACGTTG TT
	R	CAACCAAGCCTTTCCATCAT		R	GGCCAATTATTTTGCCTT GA
Set 3	F	GTTCCTGTTACCCCCGCTAT	Set 3	F	CCTGGGGAAGAGTGAAAACA
	R	ACCCTTTCACTCCTGGGTTT		R	CTTGGAAAAAGGTGCAAAGC
Set 4	F	TTCAAGCAAAATGGATGCAG	Set 4	F	ATTACGGAGGGGTGCCTACT
	R	TGGTTTTGACCAGTCCCTGT		R	GGCCAATTATTTTGCCTTGA
